# Psilocybin Therapy for Clinicians With Symptoms of Depression From Frontline Care During the COVID-19 Pandemic: A Randomized Clinical Trial

**DOI:** 10.1001/jamanetworkopen.2024.49026

**Published:** 2024-12-05

**Authors:** Anthony L. Back, Timara K. Freeman-Young, Ladybird Morgan, Tanmeet Sethi, Kelsey K. Baker, Susanna Myers, Bonnie A. McGregor, Kalin Harvey, Marlene Tai, Austin Kollefrath, Brandon J. Thomas, Dennis Sorta, Mendel Kaelen, Benjamin Kelmendi, Ted A. Gooley

**Affiliations:** 1 Department of Medicine, University of Washington School of Medicine, Seattle; 2 Department of Family Medicine, University of Washington School of Medicine, Seattle; 3 Fred Hutchinson Cancer Center, Seattle, Washington; 4 Orion Center for Integrative Medicine, Seattle, Washington; 5 Quantified Citizen, Vancouver, British Columbia, Canada; 6 Department of Psychiatry, University of Washington School of Medicine, Seattle; 7 Psychiatric Alternatives and Wellness Center, San Francisco, California; 8 Wavepaths Ltd, London, United Kingdom; 9 Yale School of Medicine, New Haven, Connecticut

## Abstract

**Importance:**

The psychological morbidity experienced by physicians, advanced practice practitioners (APPs), and nurses from working during the COVID-19 pandemic includes burnout, depression, and posttraumatic stress disorder (PTSD).

**Objective:**

To investigate whether psilocybin therapy could improve symptoms of depression, burnout, and PTSD in US clinicians who developed these symptoms from frontline clinical work during the pandemic.

**Design, Setting, and Participants:**

This double-blind randomized clinical trial enrolled participants from February to December 2022. Participants included physicians, APPs, and nurses who provided frontline care for more than 1 month during the pandemic and had no prepandemic mental health diagnoses but had moderate or severe symptoms of depression at enrollment. Participants were randomly assigned to either the psilocybin or niacin arm. Data analysis was conducted between December 2023 and May 2024 and was based on the intention-to-treat principle.

**Intervention:**

One intervention episode consisted of 2 preparation visits, 1 medication session, and 3 integration visits. At the medication session, participants received psilocybin, 25 mg, or niacin, 100 mg, orally.

**Main Outcome and Measures:**

The primary outcome was a change from baseline (preparation 1 session) to day 28 (after medication administration) in symptoms of depression as measured by the clinician-administered Montgomery-Asberg Depression Rating Scale (MADRS) used by blinded raters. The secondary outcomes were a change in symptoms of burnout (measured with the Stanford Professional Fulfillment Index [SPFI]) and symptoms of PTSD (measured with the Posttraumatic Stress Disorder Checklist for *Diagnostic and Statistical Manual of Mental Disorders, Fifth Edition* [PCL-5]).

**Results:**

A total of 30 clinicians (15 females [50%]; mean [range] age, 38 [29-60] years) participated, of whom 15 were randomly assigned to receive psilocybin and 15 to receive niacin. The mean change in symptoms of depression (MADRS scores) from preparation 1 session to day 28 was −21.33 (7.84) in the psilocybin arm compared with −9.33 (7.32) in the niacin arm, with a mean difference between arms of −12.00 (95% CI, −17.67 to −6.33; *P* < .001), a decrease in MADRS scores indicating improvement. The mean change in SPFI scores from preparation 1 session to day 28 showed a numerically larger improvement in symptoms of burnout in the psilocybin compared with the niacin arm (−6.40 [5.00] vs −2.33 [5.97]; *P* = .05) but was not statistically significant. Since the SPFI score change did not reach statistical significance, the PCL-5 score change was evaluated descriptively. The mean change in PCL-5 scores showed a numerically larger decrease in symptoms of PTSD from preparation 1 session to day 28 in the psilocybin vs the niacin arm (−16.67 [15.04] vs −6.73 [10.69]), but this difference was not statistically tested.

**Conclusions and Relevance:**

This randomized clinical trial found that psilocybin therapy resulted in a significant, sustained reduction in symptoms of depression experienced by clinicians after frontline work during the COVID-19 pandemic. The findings establish psilocybin therapy as a new paradigm of treatment for this postpandemic condition.

**Trial Registration:**

ClinicalTrials.gov Identifier: NCT05163496

## Key Points

**Question:**

Does psilocybin therapy improve symptoms of depression, burnout, and posttraumatic stress disorder in clinicians who developed these symptoms from frontline clinical work during the COVID-19 pandemic?

**Findings:**

In this randomized clinical trial involving 30 clinicians, there was a significant decrease in symptoms of depression as measured with the Montgomery-Asberg Depression Rating Scale from baseline to day 28 of psilocybin administration.

**Meaning:**

Findings of this trial indicate that psilocybin therapy is a new paradigm of treatment for postpandemic symptoms of depression associated with frontline clinical work in clinicians with moderate to severe symptoms.

## Teaser

This randomized clinical trial examines the effect of psylocibin on the level of depression, burnout, and posttraumatic stress disorder among US physicians, nurse practitioners, physician assistants, and nurses who were frontline workers during the COVID-19 pandemic.

## Introduction

The psychological morbidity experienced by physicians, advanced practice practitioners (APPs), and nurses working during the COVID-19 pandemic included burnout, depression, and posttraumatic stress disorder (PTSD). At the peaks of the pandemic, these clinicians were exposed to intense suffering, high death rates, decision-making under extreme uncertainty, prolonged work shifts, fear for their own and their families’ safety, and isolation due to self-quarantine.^1,2,3,4,5^ These experiences were psychologically challenging for some clinicians, even in the absence of preexisting mental health diagnoses.^6,7^

This pandemic-related syndrome has features of first-responder trauma, burnout, and depression.^8^ During the first peak of the pandemic, physicians, APPs, and nurses served as first responders.^8^ As the value of face masks became clear and as the vaccines arrived, these clinicians became the targets of politically motivated attacks.^9^ These incidences occurred on top of worsening burnout.^10^ While PTSD, moral distress, burnout, and depression are different constructs, there are associations between them.^11,12^

In earlier studies, psilocybin administered in the context of psychological support or therapy demonstrated improvements in individuals with major depressive disorder and treatment-resistant depression.^13,14,15^ Psilocybin therapy has also improved symptoms of depression and anxiety in patients with cancer,^16,17,18^ whose symptoms also followed a life event and involved confrontations with mortality.^19^ In this randomized clinical trial, we aimed to investigate whether psilocybin therapy could improve symptoms of depression, burnout, and PTSD in US clinicians who developed these symptoms from frontline clinical work during the pandemic.

## Methods

The University of Washington Institutional Review Board approved this trial (trial protocol in Supplement 1). Written informed consent was obtained from all participants. We followed the Consolidated Standards of Reporting Trials (CONSORT) reporting guideline.^20^

### Study Design

Between February and December 2022, this double-blind randomized clinical trial enrolled physicians, APPs, and nurses who were frontline workers during the pandemic. To be eligible, clinicians had to have more than 1 month of direct frontline clinical care experience and had to endorse at least 2 of 4 items from a COVID-19 occupational exposure index as occurring more than half of the day during that time. These items included caring for a patient critically ill with COVID-19, working longer hours to provide care to patients with COVID-19, witnessing or responding to a death from COVID-19, or caring for a patient who died without their family present due to pandemic-related precautions.^8^ Additional eligibility criteria included absence of prepandemic mental health diagnosis (self-reported), moderate or severe symptoms of depression (score ≥21) on a participant-scored version of the Montgomery-Asberg Depression Rating Scale (MADRS; score range: 0-60, with higher scores indicating worse symptoms),^21^ persistent depression symptoms for at least 6 months despite 1 or more trials of medication and/or therapy, nonpregnant, agreement to use contraception, and willingness to taper antidepressant use. Exclusion criteria included a first-degree family history of schizophrenia, bipolar disorder, or paranoid disorder; current substance use disorder; use of psychedelics within 12 months; and unstable medical conditions.

Recruitment was conducted through a study website and online newsletters. Screening for potential participants included a web-based survey; a telephone screening call; and an in-person visit with an interview, medical and psychiatric history, physical examination, laboratory tests, and an electrocardiogram.

**Figure 1.**
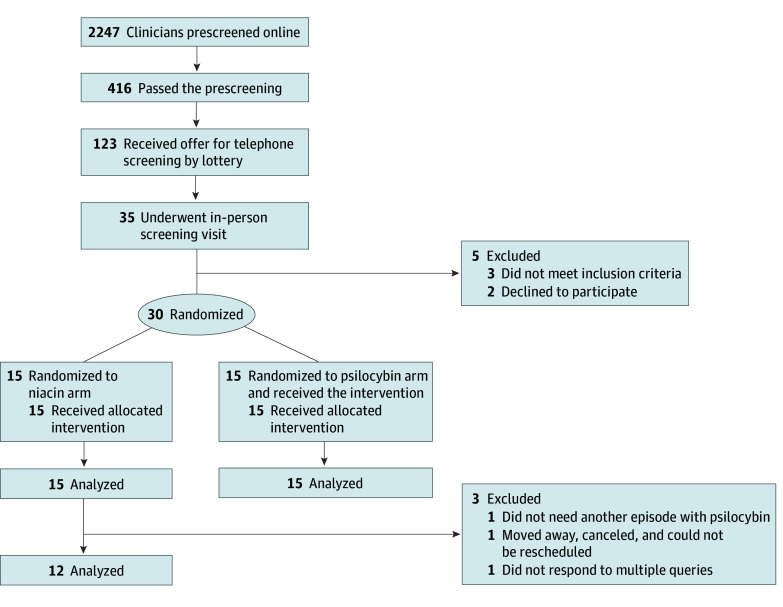
Diagram of Participant Flow Through the Trial

Randomization was performed by a University of Washington faculty member not associated with the study, who generated a random number sequence using Research Randomizer in block sizes of 6. An investigational pharmacist placed the study medication in numbered envelopes, which were dispensed in the order that medication sessions occurred. Study facilitators were given an envelope containing the study medication immediately before each medication session. The participants, facilitators, principal investigator, study coordinator, and outcome raters were all blinded to the treatment group assignment. Participants were assigned to either the psilocybin (experimental) or niacin (active placebo) arm (Figure 1). The last outcome measure from the randomized cohort was completed in August 2023.

### Intervention

One intervention episode consisted of 2 preparation sessions, 1 medication session, and 3 integration sessions or visits (eFigure in Supplement 2). The study facilitators were diverse, interprofessional, licensed clinicians with specialized training in psychedelic facilitation. Two facilitators worked with a participant through the intervention episode. One facilitator matched the participant’s professional background. Participants who self-identified as Asian, Black or African American, or Hispanic or Latinx were matched with facilitators of similar or another minoritized race and ethnicity. Race and ethnicity data were collected because a sample that approached the clinician diversity in the US would strengthen generalizability. Study-specific training was based on the EMBARK psychedelic therapy framework,^22^ including a residential retreat with experiential breathwork.^23^

At least 1 preparation session and 1 integration session were conducted in person; the rest of the sessions were provided on video. All sessions lasted 60 to 90 minutes. The 2 preparation sessions occurred on day −8 and day −1 prior to the medication (psilocybin or niacin) session. The medication session (day 0) lasted approximately 7 hours, and the 3 integration sessions occurred on days 1, 8, and 15.

A facilitator manual outlined process-specific elements for each session. For the preparation sessions, these elements included the participant’s professional story, unfinished business from their work during the pandemic, personal strengths and challenges, and intentions for the medication session. That session focused on allowing the participant to have their own experience of the medication, with minimal dialogue unless support was requested. For the integration sessions, the elements were the experience with the medication; insights or lessons from that experience; and how to take lessons forward, with an emphasis on embodied practices.^24^ Cases were reviewed in weekly meetings that included supervision.

In the experimental arm, the medication was a fixed dose of psilocybin, 25 mg, orally. In the control arm, the medication was niacin, 100 mg, orally. The medication was provided in identical white capsules (by Usona Institute) and administered with elements of secular ceremony,^25^ such as participants bringing in a photograph or keepsake. The purpose of ceremony was to reinforce the participant’s belief that they could change and to incorporate their own spirituality.

During the medication session, participants could sit in a chair or lie down on a bed, and using an eyeshade was encouraged. Both facilitators were present. The music was streamed from Wavepaths,^26,27^ played through speakers and headphones, and was a study-specific curated mix of adaptively generated and fixed musical segments with a consistent trajectory matching the pharmacologic effects of psilocybin. Facilitators instructed participants about the use of touch (eg, taking blood pressure and offering a hand for support) and elicited touch preferences.

Participant-reported measurement data were collected prior to the first preparation (preparation 1) session, the day of the medication session (day 0), and the day after the medication session (days 1, 8, 15, 28, 56, 84, and 180). Measures at the medication session were collected on paper (medication experience using Mystical Experience Questionnaire, 30 Questions [MEQ-30; 0-150, with higher scores indicating more intense symptoms]; adverse events; and suicidal ideation and behavior using Columbia Suicide Severity Rating Scale [score range varies]). Other measures were collected on a mobile secure data platform (Quantified Citizen).

### Follow-Up

Participants received check-in text messages in the evening after their medication session. In addition to the 3 planned integration sessions, additional supportive visits were available.

Participants were unblinded after they completed the day 28 questionnaires; those who received niacin could then schedule an open-label psilocybin session. Participants who were randomly assigned to the niacin arm had no further questionnaires to complete after day 28; those randomly assigned to the psilocybin arm completed questionnaires until month 6. The open-label psilocybin sessions had the same intervention structure and follow-up questionnaire schedule as the randomized psilocybin sessions.

### Outcomes

The prespecified primary outcome was a change in symptoms of depression from baseline (preparation 1 session) to day 28 (after the medication session). The primary measure was the MADRS administered by blinded raters,^28^ licensed clinicians who were trained in the MADRS for this study (provided by Valis Bioscience) and passed a qualifying examination. Participants were intentionally not given a diagnosis of depression so that they would not be obliged to report a mental health diagnosis to a licensing board. No information about study participation was entered into an electronic medical record. A certificate of confidentiality was obtained.

The prespecified secondary outcomes were change from preparation 1 session to day 28 in symptoms of burnout, measured using the Stanford Professional Fulfillment Index (SPFI; burnout subscale score range: 0-10, with higher scores indicating worse symptoms),^29^ and symptoms of PTSD, measured using the Posttraumatic Stress Disorder Checklist for *Diagnostic and Statistical Manual of Mental Disorders, Fifth Edition* (PCL-5; score range: 0-80, with higher scores indicating worse symptoms).^30^ The SPFI has 2 subscales: burnout and fulfillment. For this analysis, the SPFI burnout subscale (work exhaustion and interpersonal disengagement) and the PCL-5 are reported. Functional unblinding was assessed using 2 questions (eTable in Supplement 2).

### Statistical Analysis

The null hypothesis was that the mean change in MADRS score from the preparation 1 session to day 28 would be the same in both niacin and psilocybin groups. The alternative hypothesis was that the change in the psilocybin group would be greater than the change in the niacin group, with an assumed true effect size of 1.06 SD units based on findings in prior studies.^16,17^ With 15 participants per group, this study had 80% power to observe a statistically significant difference between groups in mean MADRS score change from baseline to day 28 (at the 2-sided significance level of .05).

Change scores were calculated as the difference between baseline and day 28 scores, with a decrease in scores indicating improvement. The difference in mean change between the 2 treatment groups was assessed with a 2-sample, 2-tailed *t* test of equal variances using the intent-to-treat principle (after testing for normality and equality of variances). A hierarchical approach was prespecified for the analysis of the primary and secondary end points in the following order: MADRS, SPFI, and then PCL-5.^31^ When one of the outcome analyses did not reach statistical significance, further outcomes were analyzed descriptively to maintain the overall type I error rate of .05. The correlation between the MEQ-30 and MADRS scores was evaluated using linear regression and summarized with a Pearson coefficient.

All reported *P* values were 2-sided, and statistical tests used a 2-sided α = .05. All analyses were performed between December 2023 and May 2024 using SAS, version 9.4 (SAS Institute Inc). No changes were made to the methods after trial commencement.

## Results

A total of 30 US clinicians were enrolled and randomized, of whom 15 (50%) were females and 15 (50%) were males with a mean (range) age of 38 (29-60) years. All participants completed all intervention sessions. Fifteen participants were randomized to psilocybin and 15 were randomized to niacin. Table 1 shows participant demographic characteristics. Participants identified their race and ethnicity from the following categories defined by the investigator: Asian (2 [7%]), Black or African American (2 [7%]), Hispanic or Latinx (1 [3%]), White (25 [83%]), or other (0). Fifteen participants (50%) were married, 3 were partnered (10%), and 12 were single (40%). The geographic region where most participants had their frontline clinical exposure during the COVID-19 pandemic was the West (25 [83%]), followed by the East (3 [10%]), Midwest (1 [3%]), and South (1 [3%]). Their frontline work exposures occurred between March 2020 and September 2021, and 29 participants (97%) were employed full-time at enrollment.

**Table 1.** Participant Demographic Characteristics

Characteristic	Participants, No. (%)		
	All (N = 30)	Niacin arm (n = 15)	Psilocybin arm (n = 15)
Age, mean (range), y	38 (29-60)	36 (30-48)	40 (29-60)
Sex assigned at birth			
Female	15 (50)	8 (53)	7 (47)
Male	15 (50)	7 (47)	8 (53)
Race and ethnicity^a^			
Asian	2 (7)	1 (7)	1 (7)
Black or African American	2 (7)	1 (7)	1 (7)
Hispanic or Latinx	1 (3)	0	1 (7)
White	25 (83)	13 (87)	12 (80)
Other^b^	0	0	0
Full-time employment at study entry	29 (97)	14 (93)	15 (100)
Profession			
MD	15 (50)	8 (53)	7 (47)
NP or PA	4 (13)	2 (13)	2 (13)
RN	11 (37)	5 (33)	6 (40)
Clinical setting during COVID-19 pandemic			
Critical care	14 (46)	8 (53)	6 (40)
Acute care	11 (37)	3 (20)	8 (53)
Emergency department	5 (17)	4 (27)	1 (7)
Counseling prior to study entry	30 (100)	15 (100)	15 (100)
Antidepressant trial prior to study	16 (53)	7 (47)	9 (60)
Tapered off antidepressant to start study	2 (7)	1 (7)	1 (7)
Used psychedelics since college	2 (7)	1 (7)	1 (7)

Abbreviations: MD, medical doctor; NP, nurse practitioner; PA, physician assistant; RN, registered nurse.

^a^ Self-identified categories using options defined by the investigator.

^b^ Other included American Indian or Alaska Native and Native Hawaiian or Other Pacific Islander.

**Figure 2.**
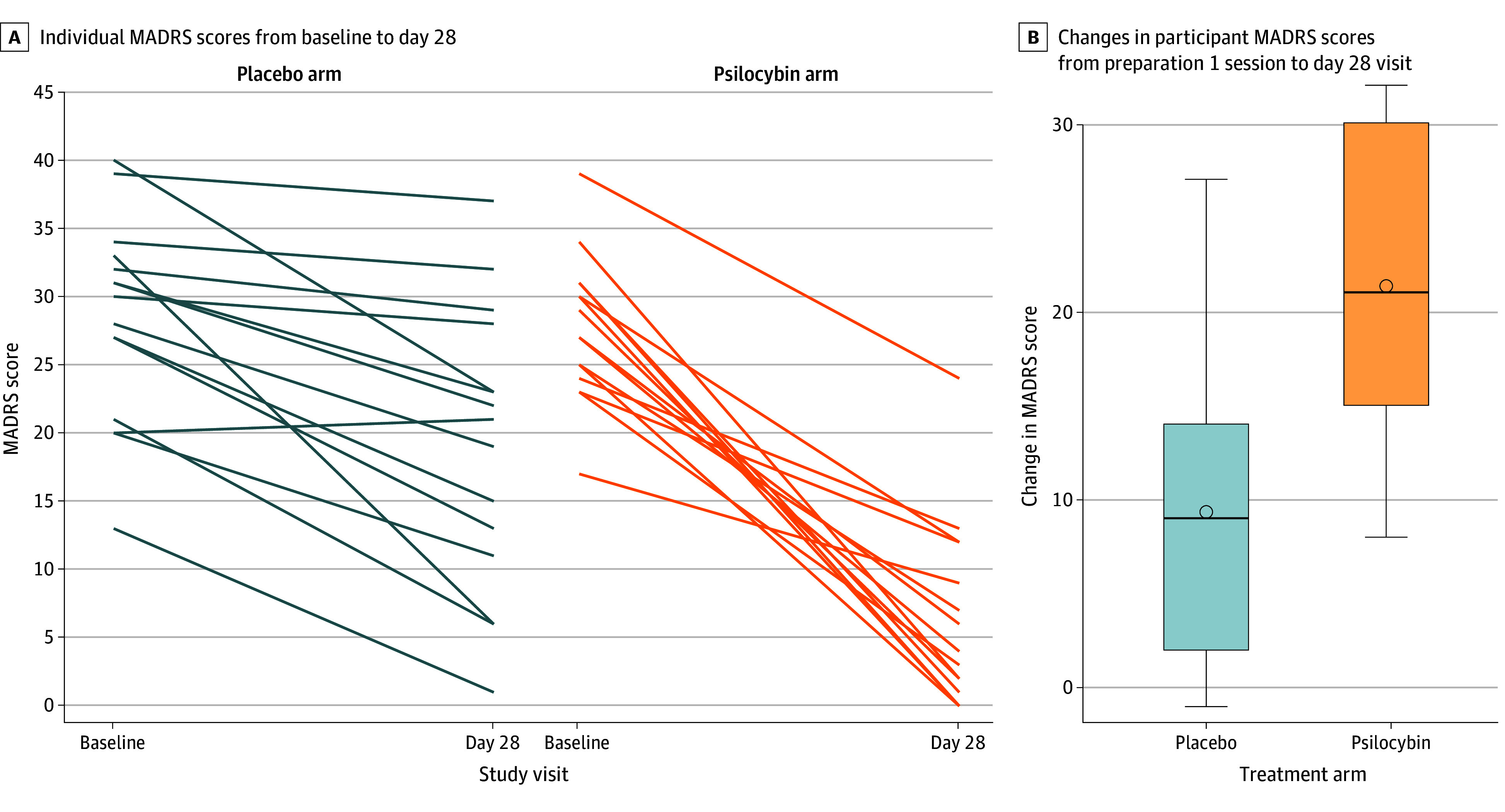
Change in Montgomery-Asberg Depression Rating Scale (MADRS) Scores for Individual Participants in Both Treatment Arms MADRS scores ranged from 0 to 10, with the higher scores indicating worse symptoms. Boxes represent the interquartile range (between the 25th and 75th percentile, the horizontal line inside boxes represents the median, and the circle inside boxes represents the mean.

**Figure 3.**
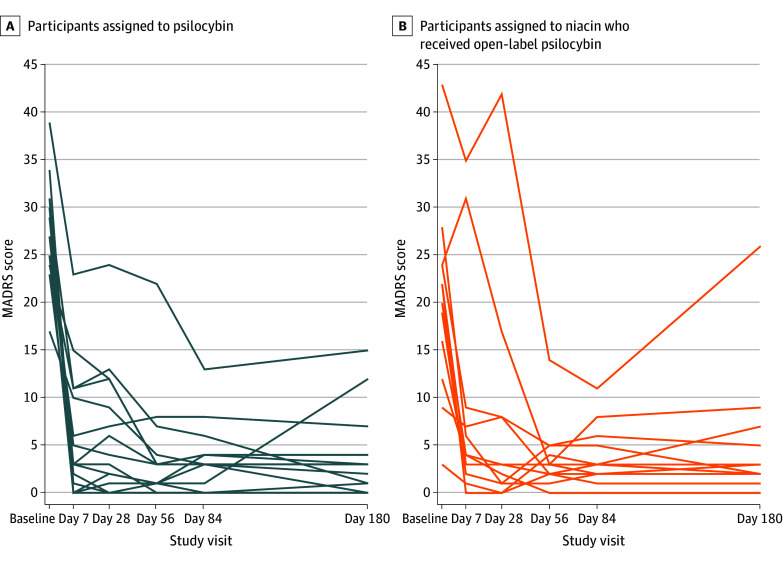
Longitudinal Symptoms of Depression From Baseline to 6 Months Montgomery-Asberg Depression Rating Scale (MADRS) scores ranged from 0 to 60, with the higher scores indicating worse symptoms.

For the primary outcome, the mean (SD) change in MADRS score from preparation 1 session to day 28 was −21.33 (7.84) in the psilocybin arm and −9.33 (7.32) in the niacin arm, with a mean difference in change scores of −12.00 (95% CI, −17.67 to −6.33; *P* < .001) (Table 2 and Figure 2). The decrease in MADRS scores (indicating improvement) in the psilocybin arm was sustained through the month 6 follow-up (mean decrease, −24.00; 95% CI, −26.87 to −21.13) (Figure 3). To put these MADRS score decreases into context, the minimum clinically important difference in change scores is 1.6 to 1.9.^32^

**Table 2.** Summary of Primary, Secondary, and Select Exploratory Outcomes

Outcome	Mean score (SD)		Mean difference (95% CI)	*P* value^a^
	Niacin arm (n = 15)	Psilocybin arm (n = 15)		
**MADRS score **				
Preparation 1	28.40 (7.39)	27.67 (5.27)	NA	NA
Day 28	19.07 (10.34)	6.33 (6.72)	NA	NA
Month 6	NA	3.67 (4.48)	NA	NA
Day 28 change^b^	−9.33 (7.32)	−21.33 (7.84)	−12.00 (−17.67 to −6.33)	<.001
**SPFI burnout score **				
Preparation 1	12.33 (3.67)	12.00 (3.87)	NA	NA
Day 28	10.00 (4.86)	5.60 (3.37)	NA	NA
Day 28 change^b^	−2.33 (5.97)	−6.40 (5.00)	−4.07 (−8.19 to 0.05)	.05
**PCL-5 score **				
Preparation 1	36.20 (13.65)	32.47 (13.91)	NA	NA
Day 28	29.47 (13.08)	15.80 (15.12)	NA	NA
Day 28 change^b^	−6.73 (10.69)	−16.67 (15.04)	NA^c^	NA
**MEQ-30 score **				
Niacin or psilocybin session score, mean (range)	15.07 (0 to 52)	129.40 (88 to 119)	NA^c^	NA

Abbreviations: MADRS, Montgomery-Asberg Depression Rating Scale (score range: 0-60, with higher scores indicating worse symptoms); MEQ-30, Mystical Experience Questionnaire, 30 Questions (score range: 0-150, with higher scores indicating more intense symptoms); NA, not applicable; PCL-5, Posttraumatic Stress Disorder Checklist for *Diagnostic and Statistical Manual of Mental Disorders, Fifth Edition* (score range: 0-80, with higher scores indicating worse symptoms); SPFI, Stanford Professional Fulfillment Index (burnout subscale score range: 0-10, with higher scores indicating worse symptoms).

^a^ *P* values were based on a 2-sample, 2-tailed *t* test with equal variances.

^b^ Day 28 change = (preparation 1 session score – day 28 score).

^c^ Not calculated due to hierarchical analysis plan.

For the secondary outcomes, the mean change in SPFI scores from preparation 1 session to day 28 showed a numerically larger decrease (indicating improvement) in burnout symptoms in the psilocybin arm compared with the niacin arm (mean [SD] score change, −6.40 [5.00] vs −2.33 [5.97]; *P* = .05). This improvement did not reach the prespecified significance level of .05, however (Table 2).

Since the first secondary outcome did not meet the prespecified significance level, the next secondary outcome, measured with PCL-5, was evaluated descriptively only. The mean change in PCL-5 scores from preparation 1 session to day 28 showed a numerically larger improvement in PTSD symptoms in the psilocybin arm compared with the niacin arm (mean score change, −16.67 [15.04] vs −6.73 [10.69]). Due to the prespecified hierarchical analysis, this difference was not statistically tested (Table 2).

The MEQ-30 scores immediately after the medication session showed a numerically greater depth of the medication experience in the psilocybin arm compared with the niacin arm (mean [range] score, 129.40 [88-119] and 15.07 [0-52]). Higher MEQ-30 scores showed a modest correlation with improvement in MADRS scores from preparation 1 session to day 28 (*R* = 0.701).

No serious adverse events occurred. Other adverse events were managed without medical intervention except for 1 instance of nausea in the psilocybin group. On the day of the psilocybin administration, other adverse events occurred, such as mild nausea (4 [27%]), mild headache (4 [27%]), mild tachycardia (2 [13%]), and hypertension (mild, 6 [40%], moderate, 8 [53%], or severe, 1 [7%], which resolved in <20 minutes without medical treatment). There were no episodes of psychosis or attempts to leave the room without permission. For the niacin sessions, 1 participant experienced a mild headache. After day 0, there were no episodes of thought distortion (derealization or depersonalization) or perceptual disturbances. The most serious psychiatric adverse event involved 1 participant on day 28 after receiving open-label psilocybin; the individual had transient thoughts that they might be “better off dead,” without suicidal intention or plans; these thoughts did not recur.

The functional unblinding questions showed that just after they swallowed the capsule, 100% of participants said they did not know whether they received psilocybin or niacin. At the end of the session, however, 100% stated they knew—and were correct (eTable in Supplement 2).

Illustrative comments from the preparation sessions included the following: “I feel more disposable than a used COVID-19 swab”; “It got to the point where I felt like I was participating in the torture of people”; and “I found myself waking up in the middle of the night and logging on to Epic to see if anyone I’d sent home had come back and died or gone to the ICU [intensive care unit].” Illustrative comments from the integration sessions were as follows: “I’m reaching out and leaning on others…and trusting that the people around me can support me. I don’t have to carry it all by myself”; “I’m learning to just embrace dystopia…you have to adapt”; and “I was shown my tendency to be a superhero…[during my trip I received] messaging that ‘It’s not about you’—that was done with grace, love, and accountability.”

Of the 15 participants in the niacin group, 12 (80%) received an episode of open-label psilocybin therapy. These open-label episodes had preparation, medication, and integration sessions and follow-up questionnaires, as in the randomized psilocybin arm. In these open-label episodes, the mean (SD) baseline MADRS score was 20.17 (10.11), and the mean change in MADRS score from preparation 1 session to day 28 was −12.83 (95% CI, −18.29 to −7.38) (Figure 3).

Employment changes over the course of the trial follow-up were notable. Because most participants assigned to the niacin group went on to receive open-label psilocybin, these results are reported for the entire group. During their follow-up, 21 participants (70%) reported a change in their work role or full-time equivalent status that substantially changed their responsibility or institution; 8 (27%) remained in the same position; and 1 (3%) remained unemployed. No one left the health care field.

## Discussion

To our knowledge, this trial is the first to demonstrate the utility of psilocybin therapy in the treatment of physicians, APPs, and nurses who developed moderate to severe symptoms of depression in the course of frontline work during the COVID-19 pandemic. Biologically, psilocybin is a partial agonist of the 5-HT2A receptor, with downstream effects of inducing neuroplasticity, which likely underlies these benefits.^33,34,35^ Therapeutically, psilocybin in this study was administered within the context of preparation, medication, and integration sessions by specially trained clinicians.^36,37^ These facilitators used session-specific protocols that emphasized the possibility of change, allowing emotions typically avoided, in-the-moment unfolding of the medication session, insights emerging from that experience, and cultivation of practices to bring those insights into daily life. The statistically significant difference between the psilocybin and niacin arms, both of which had the same counseling, attests to how much psilocybin added to the therapeutic intervention.

The results for the primary outcome showed a statistically significant improvement in symptoms of depression (as measured by the MADRS) for participants in the psilocybin arm, which was sustained for most of the participants for 6 months. This 21-point decrease in MADRS scores is striking when a 6- to 9-point change in the MADRS is considered clinically meaningful.^38^ The change in symptoms of burnout did not reach statistical significance, but this small trial may have been inadequately powered for this outcome. A recent systematic review of interventions for physician burnout tested in randomized clinical trials found numerical changes that were “unlikely [to] result in meaningful changes in clinical burnout”^39^^(p249)^; in contrast, a number of participants described their study experience as life-changing. Furthermore, while the significance of the change in PTSD symptoms was not analyzed due to the hierarchical analytic plan, the 16-point decrease in PCL-5 scores for the psilocybin arm was well above the 10-point decrease considered clinically meaningful.^40^

This trial builds on past studies showing that psilocybin therapy was effective for depression and treatment-resistant depression in individuals with much longer durations of illness and often multiple regimens of antidepressants. By contrast, participants in this study had no prepandemic mental health history other than moderate to severe symptoms of depression, burnout, and PTSD. The efficacy of psilocybin in this study is notable given that 100% of the participants had previously tried counseling and more than 50% had tried an antidepressant. Their rapid and sustained response indicates that psilocybin therapy is a new paradigm of treatment for the postpandemic depressive symptoms in clinicians.

Although the trial did not include an arm of psilocybin without therapy, the issues that emerged suggest a complex psychological landscape that would be difficult for any individual to navigate alone. Clinicians described a sense of betrayal by health systems, leaders, and colleagues; guilt from feeling that they had not been able to do enough; and grief from witnessing innumerable deaths and suffering. The question that repeatedly came up, in different forms, was “Do I matter?” What psilocybin-assisted therapy did, when it was successful in this trial, was enable participants to take some time amid the urgency of their professional and personal lives to feel all of their feelings, find some perspective on their recent past, and come to terms with what they were unable to do—and what they were able to accomplish—for patients, families, colleagues, and society. Following their experience, many participants were able to locate their own resources, give themselves permission to take care of themselves, and start to reconstruct the meaning that their work still holds. While most participants intentionally made major changes to their clinical work during their participation in the trial, none left health care altogether.

### Limitations

This study has limitations. Because it was a small trial, its findings might not be generalizable. Many more clinicians indicated interest (2247) than could be enrolled (30), and while we selected participants randomly at each step of recruitment, unknown biases may be present. Additionally, the participants and facilitators, all of whom reported not knowing which medication was given at the moment of ingestion, found that after 2 hours they could distinguish niacin from psilocybin with 100% precision. Functional unblinding is an issue for all studies involving psychedelic therapies; in this study, it was mitigated with a primary outcome assessed by blinded raters who had no contact with the therapist team. This, however, may not address other effects that unblinding could have.

## Conclusions

In this randomized clinical trial, psilocybin therapy was associated with a significant and sustained reduction in symptoms of depression experienced by physicians, APPs, and nurses after their frontline work during the COVID-19 pandemic. The results establish psilocybin therapy as a new paradigm of treatment for this postpandemic condition and add to the evidence of psilocybin therapy for depression. Further research is warranted to assess the role of psilocybin therapy in meeting the well-being challenges faced by clinicians in the workplace.

## Notes

Supplement 1.
Trial Protocol

Supplement 2. **eTable.** Functional Unblinding Questions **eFigure.** Study Flow Diagram

Supplement 3.
Data Sharing Statement
