# Error in the Byline

**DOI:** 10.1001/jamanetworkopen.2024.59278

**Published:** 2025-01-13

In the Original Investigation titled, “Psilocybin Therapy for Clinicians With Symptoms of Depression From Frontline Care During the COVID-19 Pandemic: A Randomized Clinical Trial”^1^ published December 5, 2024, one of Ladybird Morgan’s academic degrees was omitted from the byline. The name should appear as follows: Ladybird Morgan, RN, MSW. The article has been corrected.

## References

[1] Back AL, Freeman-Young TK, Morgan L, . Psilocybin therapy for clinicians with symptoms of depression from frontline care during the COVID-19 pandemic: a randomized clinical trial. JAMA Netw Open. 2024;7(12):e2449026. doi:10.1001/jamanetworkopen.2024.49026 39636638 PMC11621983

