# Errors in the Author Affiliations

**DOI:** 10.1001/jamanetworkopen.2025.25303

**Published:** 2025-07-10

In the Original Investigation titled “Psilocybin Therapy for Clinicians With Symptoms of Depression From Frontline Care During the COVID-19 Pandemic: A Randomized Clinical Trial,”^1^ which was published on December 5, 2024, several author affiliations were incorrect. The affiliation for Dr Back, Ms Freeman-Young, and Ms Morgan should be the Department of Medicine, University of Washington School of Medicine, Seattle. The affiliation for Dr Sethi should be the Department of Family Medicine, University of Washington School of Medicine, Seattle. The affiliation for Drs Tai, Kollefrath, and Thomas should be the Department of Psychiatry, University of Washington School of Medicine, Seattle. The article has been corrected.
