# Supplementary material for: Psilocybin Therapy for Clinicians With Symptoms of Depression From Frontline Care During the COVID-19 Pandemic: A Randomized Clinical Trial
Source: JAMA Netw Open. 2024 Dec 5;7(12):e2449026. doi: 10.1001/jamanetworkopen.2024.49026 (PMC11621983; doi:10.1001/jamanetworkopen.2024.49026)
Supplement: Supplement 1. — Trial Protocol [file jamanetwopen-e2449026-s001.pdf]

Study Title:
A randomized, placebo-controlled trial of psychedelic-assisted psychotherapy with single dose psilocybin for frontline clinicians experiencing COVID-related symptoms of depression and burnout

Principal Investigator:
Anthony Back MD
University of Washington

IND/IDE Sponsor:
Anthony Back MD

Protocol number:
BACK001

Revision
July 18, 2022
Version 1.16

Funded by:
Steven and Alexandra Cohen Foundation

Table of Contents

1. Introduction 3
2. Study Design and Endpoints 16
3. Research Materials and Measures 18
4. Participant Inclusion and Exclusion Criteria 22
5. Study Agent 25
6. Study Procedure and Schedule 26
7. Payments for Participation (Economic Considerations) 32
8. Assessment of Safety 32
9. Statistical Considerations 36
10. Ethics/Protection of Human Subjects 37
11. Data Handling and Record Keeping 38
12. Publication and Data Sharing Policy 39
13. Study Administration and Leadership 39
14. Conflict of Interest Policy 39
15. References 39

1. Introduction

1.1. Statement of Purpose.

We aim to investigate the effects of a single dose of psilocybin, delivered in the context of pre- and post-dose psychotherapy, on symptoms of depression and burnout suffered by healthcare clinicians as a result of frontline work in the COVID pandemic.

1.1.1 Aim 1:

To assess short- and longer-term effects of psilocybin-assisted psychotherapy (PAP) on symptoms of depression experienced by physicians and nurses with frontline work exposure in the COVID pandemic.

Hypothesis 1.1: Compared to active placebo, PAP will result in short term improvement in symptoms of depression 1 day and 1 week after the psilocybin dose session.

Hypothesis 1.2: Compared to active placebo, PAP will result in longer term improvement of symptoms of depression 4 weeks after the medication dosing session. The primary outcome will be a comparison between the psilocybin 25 mg vs control groups of a combination of depression symptoms measured at 4 weeks post medication dose session.

1.1.2. Aim 2:

To explore short- and longer-term effects of psilocybin-assisted psychotherapy (PAP) on symptoms of burnout experienced by physicians and nurses with frontline work exposure in the COVID pandemic.

Hypothesis 2.1: Compared to active placebo, PAP will result in short term improvement in symptoms of burnout 1 day and 1 week after the psilocybin dose session.

1.2. Background and Rationale

The COVID19 pandemic is associated with significant psychological morbidity for physicians and nurses with frontline work exposure. The experiences of these physicians and nurses include multiple, intense exposure to patients who are suffering and dying, stress related to clinical decision making under extreme uncertainty especially at the beginning of the pandemic, prolonged periods of work and vigilance leading to physical and emotional exhaustion, fear for their own safety and their families' safety, and often extended separations from family related to self-quarantining because of risk to their families. In addition, stress from preparing for crisis standards of care that would require clinicians to make rationing decisions, moral injury from feeling that they were prevented from doing their best work, and disillusionment from layoffs that many health systems made to protect their financial status have compounded these stressors. Interviews of physicians and nurses published in the lay press have emphasized that 'we'll never be the same'[1-3] and also that psychological morbidity occurs in physicians and nurses with no prior history of mental health issues or treatment.[4-6]

The psychological morbidity for frontline physicians and nurses includes symptoms of depression, anxiety, burnout and post-traumatic stress. Front-line clinicians (physicians, nurse practitioners, physician assistants, nurses) in the COVID pandemic are experiencing high levels of psychological sequelae related to distress experienced in direct clinical care. In a study of health care workers in Wuhan, China, "a considerable proportion of participants reported symptoms of depression (634 [50.4%]), anxiety (560 [44.6%]), insomnia (427 [34.0%]), and distress (899 [71.5%])."[7] In addition, "frontline health care workers engaged in direct diagnosis, treatment, and care of patients with COVID-19 were associated with a higher risk of symptoms of depression (OR, 1.52; 95%CI, 1.11-2.09; P = .01), anxiety (OR, 1.57; 95%CI, 1.22-2.02; P < .001), insomnia (OR, 2.97; 95%CI, 1.92-4.60; P < .001), and distress (OR, 1.60; 95%CI, 1.25-2.04; P < .001).[7] A qualitative study found that "The intensive work drained health care providers physically and emotionally."[8] A physician suicide in the US to date attributed to medical work in

hospitals overwhelmed with COVID is notable for the complete lack of any depressive history in a physician who contracted COVID herself, returned to work, was hospitalized for severe depressive symptoms, and took her own life days after discharge.[9]

These psychological symptoms of stress are somewhat different than existing DSM classifications of depression and anxiety disorders. An unpublished but publicly released national study of health care workers demonstrates that symptom severity is clearly related to intensity of exposure.[10] Stressor frequency scores correlated significantly with PCL5 scores ($R=.57$, $p<1e\text{-}8$), PHQ scores ($R=.35$, $P<.001$), ISI scores ($R=.38$, $p<1e\text{-}4$), and GAD7 scores ($R=.39$, $p<.001$), likelihood of staying in current occupation ($R=-.39$, $p<1e\text{-}4$), and trouble doing usual work ($R=.33$, $p<.001$). 51% of HCW and 44% of FR indicated decreased likelihood of staying in their current occupation. PCL5 scores substantially mediated the association between stress frequency scores and work function impairment.

The implications for the medical workforce that the US needs for the continuing pandemic are substantial. An unpublished but publicly released survey of clinicians carried out by the Larry A Green center and the Primary Care Collaboration reported that 19% of survey respondents reported that clinicians in their practice had retired early because of COVID-19 or were planning on it.[11]

Existing interventions for first responders, treated in disaster situations with some similarities to COVID have shown no impact on these psychological sequelae. A 2012 systematic review concluded that "the literature is startlingly sparse and is not sufficient for evidence-based recommendations for first responders.[12] A more recent 2020 systematic review of psychological interventions for first responders was also unable to find enough evidence to name any best practices.[13] Thus while many physicians and nurses are already turning to existing treatments with antidepressant medications and psychotherapy, there is as yet no data supporting efficacy of these treatments—and data suggests that physicians in particular are not willing to be treated with antidepressants. In a study of over 700 resident physicians, where 17% scored positive for depression, only about a quarter of those scoring positive were receiving treatment (either prescribed through formal treatment, informally through a colleague, or self-prescribed) for depression.[14] Physicians have a negative incentive to seek any type of mental health care as many state licensing agencies require physicians to report mental health treatment and make this information publicly available.

Psychedelic-assisted psychotherapy is a new, promising form of treatment for depression and other mental health disorders. Although psychedelic medications were widely used by psychiatrists in the 1950s and 1960, and considered a 'major breakthrough' in the treatment of depression and other mental health conditions, changes in federal legislation banning the use of psychedelics heavily impacted the use of psilocybin, LSD, and other medications in 1970 with the Controlled Substances Act.[15] Due to these changes, the available studies are from the 1950s and 1960s and these studies often did not have the level of scientific rigor or gather the types of data that is commonplace today. However, self-reported user experience indicates that psychedelics are quite safe. The 2010 National Survey on Drug Use and Health, reporting a survey of 57,873 randomly selected Americans about their drug history , found that about 32 million Americans (or 17% of adults between 21 and 64) have used any psychedelic drugs at least once in their lifetimes.[16] (The survey is limited as participants were not asked when they had used psychedelic medications.)

A new wave of studies, starting in 2018, have shown that psychedelics, given in the context of carefully designed psychotherapy, can improve symptoms of depression for weeks and in some people, years. Randomized clinical trials of psychedelic-assisted psychotherapy, mostly using pharmaceutical grade synthesized psilocybin (the psychedelic in 'magic mushrooms') have shown strikingly positive findings, and led to a FDA designation for psilocybin as a 'breakthrough treatment'.[17] Earlier this year a randomized study of psilocybin-assisted psychotherapy for major depression (not treatment-resistant depression) showed strongly positive results.[18] "The mean (SD) GRID-HAMD scores at weeks 1 and 4 (8.0 [7.1] and 8.5 [5.7]) in the immediate treatment group were statistically significantly lower than the scores

at the comparable time points of weeks 5 and 8 (23.8 [5.4] and 23.5 [6.0]) in the delayed treatment group. The effect sizes were large at week 5 (Cohen d = 2.2; 95%CI, 1.4-3.0; $P < .001$) and week 8 (Cohen d = 2.6; 95%CI, 1.7-3.6; $P < .001$)." Moreover, the antidepressant effect was immediate—noted by participants on the first day after undergoing the supervised psilocybin ingestion.

Because major depressive disorder typically has a different antecedent history than specific trigger of COVID pandemic work exposure suffered by physicians and nurses, another group of patients with depressive symptoms following a trigger is a useful comparison—patients with cancer. In two randomized studies of patients with cancer—many with incurable cancer—suffering symptoms of depression and anxiety, treatment with psilocybin-assisted psychotherapy resulted in very significant improvements in symptoms after a single treatment sequence of preparation psychotherapy, medication dosing with supervision, and follow up integration psychotherapy. In the study conducted by Ross at NYU[19] (a consultant to this proposal), the results indicated "For each of the six primary outcome measures (HADS T, HADS A, HADS D, BDI, STAI S, STAI T), there were significant differences between the experimental and control groups (prior to the crossover at 7 weeks post-dose 1) with the psilocybin group (compared to the active control) demonstrating immediate, substantial, and sustained (up to 7 weeks post-dosing) clinical benefits in terms of reduction of anxiety and depression symptoms."

The scientific mechanism for these findings is that treatment with psilocybin-assisted psychotherapy occasions a kind of 'reset' in ruminative, self-critical inner narratives—patterns which can be completely changed with psychotherapy and psilocybin. One of the leading researchers in the field, at Imperial College London writes in a 2020 review in Cell that "We suggest one way of looking at the difference between them is that current medicines [e.g., SSRIs] suppress symptoms in a similar way that insulin suppresses hyperglycemia in diabetes. Standard antidepressants protect against the stressors that lead to and perpetuate depression, but don't directly access and remedy underlying biopsychosocial causes. In contrast, psychedelic therapy harnesses a therapeutic window opened up by the brain via the effects of the drugs to facilitate insight and emotional release and, with psychotherapeutic support, a subsequent healthy revision of outlook and lifestyle."[15]

At the neurological level, psychedelics appear to open 'a therapeutic window' by dysregulating activity in brain circuits and systems that encode habits of thought and behavior. Psilocybin, perhaps the most intensively studies of the psychedelics, is a 5-Hydroxytryptamine 2A (5-HT2A) receptor agonist.[20] The 5-HT2A receptor is maximally expressed in the cerebral cortex, and thus treatment with psilocybin dysregulates cortical function that is associated with predictive processing that the brain normally undertakes to enable an individual to make decisions throughout the day. These psilocybin-induced changes in predictive processing result in both the altered perceptions often reported by individuals in the first 6 hours after ingesting psilocybin and more importantly, altered cognitions that can persist after the psilocybin effect has worn off—and these new cognitions are insights that are reinforced and articulated during the follow up integration psychotherapy.[15]

Psychedelic-assisted psychotherapy with psilocybin has the evidence profile best suited to treat clinician COVID-related distress of all tested psychedelics because of its documented efficacy with symptoms of depression**.** To be fair, there is limited evidence for psilocybin-assisted psychotherapy to address symptoms of post-traumatic stress disorder, and a Phase 3 study is currently underway testing the efficacy of MDMA-assisted psychotherapy for PTSD from other causes.[21] However, the MDMA Phase 3 study does not target frontline clinicians, and is not designed to study clinician COVID-related distress. The suitability of MDMA for COVID-related distress is less clear in that no recent evidence exists to support the efficacy of MDMA-assisted psychotherapy for existential distress. There are older studies of MDMA for cancer patients, but those studies were conducted before validated measures of existential distress or post-traumatic stress were available,[22] and ongoing studies have not been reported. Overall, because frontline clinician distress appears to contain major components of anxiety, depression, and existential distress, at this point in the state of the science, **psilocybin is the psychedelic medication with the evidence profile best suited for COVID-related distress**.

Psilocybin will be used in a proven pre- and post-dose psychotherapeutic process defined by prior research conducted by Griffiths and Ross.[19,23] In these studies, psychotherapy involved 2 trained therapists for each study participant, and included Preparation sessions conducted in for 6-8 hours total (in 3 sessions) prior to a Psilocybin Dose session, followed by Integration sessions for 6-8 hours (again in 3 sessions). These studies reported a few toxicities, and no severe adverse events. This psychotherapeutic context of Preparation/Psilocybin Dose/Integration was extremely well tolerated and resulted in improvements in anxiety, depressive symptoms, and existential distress that were statistically significant and clinically meaningful. Cancer patients who participated in the Griffith and Ross studies described in qualitative interviews how their existential issues came to have different meanings after their participation in the study in ways that allowed them to cope better, face death with less fear, and experience better quality of life.[24] Because this proposal involves psychedelic-assisted psychotherapy with a new indication and new target population for psychedelic-assisted psychotherapy, the scientific approach most likely to yield valuable results would use a psychotherapeutic process that has been proven.

1.3. Potential Benefits and Risks

1.3.1 Benefits

Subjects in this study may experience relief of symptoms from depression and burnout, but it is unknown whether these short term benefits will translate into significant, long-term benefits.

1.3.2 Potential Benefits

Participating physicians and nurses may benefit from the medical and psychiatric evaluations that they receive from participating in the study, which will be more evaluation that most of them receive during their routine work. Some participants may benefit by learning about depression, mood disorders, and burnout, and by gaining insight about their symptoms through participation in the study.

The medical knowledge gained by this study may contribute to the understanding of clinician depression and burnout and could benefit future clinicians affected by symptoms of depression and burnout as a result of the work during the COVID pandemic.

In addition, knowledge gained by this study could lead to development of a novel therapy for clinicians who have been severely affected by their clinical work in the COVID pandemic.

1.3.3. Risks to Participants

1.3.3.1. Risk of the experimental medication (synthesized psilocybin).

According to the most recent and comprehensive review, serotonergic psychedelic medications “are generally considered to be physiologically safe molecules” that chiefly alter consciousness.[25] Recent reviews and searches of the literature indicate that psilocybin is not associated with harm or damage to any organ or system in the body.[26] Psilocybin can produce changes in blood pressure and heart rate, but these changes are not as strong or consistent as those seen after psychostimulants (amphetamines), and sometimes these changes only occur at one time point.[25] More consistently, psilocybin can produce rapid and intense changes in mood including periods of anxiety or panic. Both these physiological and psychological effects are transient and do not last beyond the duration of drug effects.

Researchers studying the effects of 30 mg psilocybin took precautions similar to the ones we will use in this study, and no reactions requiring pharmacological intervention occurred.[23,27]

1.3.3.1.a. Safety and Pharmacology of Psilocybin (mushroom-derived, and synthesized)

Studies in humans and nonhuman animals indicate that psilocybin has very low toxicity.[28,29] The LD_{50} ranged from 285mg/kg in rats and mice.[30] The maximum tolerated dose of psilocybin in humans has not been defined or established. The best estimate of a lethal dosefor pure psilocybin in humans is about 19 grams. The full dose of psilocybin that will be administered in this study is 1/100 times of the established LD_{50} in nonhuman animals and in humans. A Phase I trial of psilocybin completed in 2019 at oral doses of up to 0.6 mg/kg demonstrated no serious adverse events.[31] Psilocybin is not associated with disease or damage to any organ or system.[29] More commonly, damage or disease to organs (as renal failure) is associated with mistakenly consuming poisonous mushrooms under the belief that they are psilocybin-containing mushrooms.[32]

Three cases of death possibly related to the direct toxic effects of Psilocybe mushrooms (the natural source of psilocybin) have been reported in the world literature. One fatality occurred in a post-cardiac transplant patient who ingested Psilocybe mushrooms.[33] It is not known however whether psilocybin was the proximal cause of death orwhether death occurred secondary to the cardiac stimulant phenethylamine, which is also present in the mushrooms. In the French literature, another fatality linked to Psilocybe mushroom use was associated with far higher blood concentrations of the active metabolite of psilocybin than would be expected with the doses proposed in this study.[34] A final fatality from Psilocybe mushroom ingestion was mentioned in a 1961 review but was poorly characterized, with no psilocin concentrations were reported.[35]

In a review of other adverse effects of psilocybin from Psilocybe mushrooms, an additional 10 individuals were reported to have experienced accidental deaths associated with Psilocybe mushroom ingestion (van Amesterdam et al. 2012). In most cases, these accidental deaths resulted from falls from buildings. Given the documented history of use of *Psilocybe* mushrooms in a variety of societies and cultures, that there are only 3 known fatalities at best indirectly linked to the physiologic effects of mushroom ingestion is an argument for the general safety of this natural product. We believe that the use of synthesized psilocybin will decrease the risk of cardiac toxicity by avoiding any additive or synergistic effect of phenylethylamine. Further, a recent review of the potential harm of the ingestion of *Psilocybe* mushrooms by the Dutch medicinal advisory board found that the risk of acute or chronic toxicity was low (van Amsterdam, Opperhuizen, and van den Brink 2011).

The source of synthesized psilocybin used in this study is the Usona Institute.[36] The Usona Institute is a non-profit medical research organization founded in 2014, and has developed synthesized psilocybin for use in investigator-initiated studies and Usona Institute-sponsored studies (currently underway for Major Depressive Disorder). The Usona Institute synthesized psilocybin has been used in a variety of studies, including open-label, dose-escalation studies in healthy volunteers, patients with obsessive compulsive disorder, alcohol use, nicotine use, and in randomized studies involving patients with depression and cancer.[19,37-40]

To date, over 2000 participants have received synthesized psilocybin under controlled conditions in scientific studies with no reports of the occurrence of a significance of adverse events deemed to be associated with drug administration. The most commonly reported adverse events from the scientific literature are psychological in nature and include the induction of negative emotional states and paranoid/delusional thinking during psilocybin sessions, as well as far less frequent reports of Hallucinogen Persisting Perception Disorder (HPPD).[26] A review of studies conducted worldwide between 1999 and 2008 identified only

one subject (out of 110) who experienced any persistent perceptual symptoms associated with HPPD and these symptoms were mild, brief, and resolved within three days of psilocybin exposure.[41] Rates of prolonged psychiatric symptoms of any kind following psilocybin exposure in healthy study participants are estimated to be 0.08-0.09%. As in the phase 2 studies, common physical adverse events associated with psilocybin administration include increased BP and heart rate, nausea, and headaches. In the phase 2 studies of cancer related anxiety and depression no cases of HPPD were identified and no participants developed any symptoms of paranoia or anxiety that required pharmacological interventionor anything more than reassurance from session facilitators.[19,23] In a survey of 1993 individuals in the context of unsupervised, illicit use of psilocybin-containing mushrooms, 11% put self or others at risk of physical harm. Of the respondents, 2.6% behaved in a physically aggressive or violent manner and 2.7% received medical help. Of those whose experience occurred >1 year before, 7.6% sought treatment for enduring psychological symptoms. 3 cases appeared associated with onset of enduring psychotic symptoms and 3 cases with attempted suicide.[42]

To date, there have no reports of physical harm in patients who have received psilocybin under controlled conditions, and no fatalities have been associated with its use in a controlled clinical trial. The difference in the side-effect profile between the unknown doses of psilocybin-containing mushrooms consumed in an uncontrolled setting and dose-controlled medically prepared oral psilocybin consumed in a controlled setting is thought to be in part doe the focus on the set and *setting* of the experience. The *set* refers to the emotional/cognitive/behavioral state/mindset and expectations of study participants just prior to psilocybin exposure and *setting* refers to the physical environment in which the exposure occurs.[43]

There are no confirmed reports of an overdose of synthesized psilocybin. In the United States, use of chemically synthesized psilocybin does not occur.[44] Although psilocybin in the form of mushrooms is sometimes used non-medically, medical emergencies due to psilocybin mushrooms are very rare (psilocybin is mentioned in only 0.1% of drug-related emergency department visits).[45] It can be anticipated that an overdose of psilocybin might present in a manner similar to "serotonin syndrome". Removal of any residual gastric drug, supportive care, and cautious administration of a serotonin antagonist such as risperidone are reasonable interventions; however, to date no cases have described similar responses with respect to psilocybin, and the doses of psilocybin that might provoke these physiological effects would be many times greater than even the highest doses used in early human studies.

1.3.3.1.b. Common side effects of psilocybin

Common acute side effects of psilocybin are almost all psychological, and include anxiety, changes in thought (experiencing thinking speeding up or slowing down), changes in motion perception, changes in time perception (time slowing down or speeding up), depersonalization (feeling as if one is "outside oneself"), derealization (feeling as if the world is unreal or as if one is "in a dream"), dizziness, fatigue, impaired concentration, inattention, mood lability (rapid and sometimes profound changes in mood), nausea, nervousness, parasthesias (strange bodily sensations or feelings), perceptual alterations (general), altered time perception, alteration in visual perception (distortions, illusions and imagery seen with eyes open or closed), and unusual thought or feelings about the self or the world.[25,41,46] Most of these effects are acute and last no longer than six hours. For the most part, people receiving psilocybin were able to interact, and maintained insight concerning the nature and source of their experience. However, some participants occasionally exhibited paranoid ideation or temporarily lost insight into the experimental situation.[41,46]

Psilocybin produces mild sympathetic system activation. Physiological effects include pupillary dilation and detectable but moderate increases in blood pressure or heart rate .[46] Researchers sometimes detected changes in blood pressure and heart rate only at one point in time, as sixty minutes after drug administration.[25] These effects are transient and are generally gone approximately six hours after drug administration. People in previous human studies have tolerated doses of psilocybin equal to or greater than the doses employed in this study. Researchers studying the effects of 30 mg psilocybin took precautions similar to the ones we will use in this study and no reactions requiring pharmacological intervention occurred.[27]

1.3.3.1.c. Acute side effects

Anxiety. The most likely potential adverse effect of psilocybin is anxiety, or possibly panic, delusion,and cognitive impairments, particularly at higher doses (> 25 mg po) during the period of acute drug action. In recent studies of psilocybin in healthy humans conducted over the past 15 years, no adverse reactions other than the expected occasional episodes of fear or anxiety during the time of drug effect have been reported.[25,29] Such transient episodes of fear or anxiety respond well to reassurance and have not required pharmacological intervention. Some individuals have been reported to develop paranoid and psychotic states while under the influence of psilocybin.

Insomnia. Although rare, insomnia following psilocybin administration has been reported in the literature; it has been described as self-limiting and responded well to reassurance from the study team.[41] In the event that a participant experiences insomnia, a study physician may initially prescribe a sleep aid (i.e. diphenhydramine or hydroxyzine) and if the initial reassurance, education about sleep hygiene and pharmacological intervention was unsuccessful, a one-time dose of short-acting benzodiazepine (specifically lorazepam) and/or zolpidem will be considered.

1.3.3.1.d. Prolonged side effects

There is the possibility ofprolonged adverse psychological reactions, such as psychosis and depression. It is generally believed that individuals with previous psychiatric conditions, particularly psychosis or mania, are at an increased risk for hallucinogen-induced psychosis.[29] Thus, psilocybin administered to a participant with a history of psychosis or mania could precipitate enduring psychiatric illness such as schizophrenia. This risk is very low, but cannot be excluded completely.[47] For this reason, all potential participants with personal or family histories of Schizophrenia, Psychotic Disorder, or Bipolar I or II disorder are to be excluded from participation. Nevertheless, although the risk is believed to be minimal, it is possible that some participants may develop a brief intense psychological reaction while under the influence of psilocybin.

The low rate of late psychological symptoms is consistent with a summary of such effects from 110 psilocybin research participants reported in a meta-analysis conducted by Studerus et al in their laboratory.[41] In that report, 7 participants endorsed negative changes in psychological well-being, but only 1 participant (0.9%) reported a level of distress sufficient for him to contact the researchers. Those symptoms resolved after a few sessions with an experienced psychotherapist.

Investigators at the Johns Hopkins Center for Psychedelic and Consciousness Studies recently completed an internet survey of hallucinogen users who reported having a challenging experience after taking psilocybin mushrooms.[42] The rates and severity of both acute and enduring problems shown in the survey are notably higher than those observed in

laboratory research studies involving administration of high doses of psilocybin to carefully screened, well-prepared, and closely monitored volunteers. This observation appears to support the efficacy of the standard screening, preparation, monitoring, and follow-up procedures designed to minimize the possibility of any enduring problems.

1.3.3.2. Potential risks of Niacin (active-placebo-control agent)

A participant's symptoms of depression or burnout could change or perhaps worsen during the course of the study, as symptoms might change if that individual were not participating in the study. However, there is no reason to believe that niacin as an active-placebo-control would exacerbate symptoms of depression or burnout. Niacin has expected physiological side effects that make it well suited to be used as an active placebo, including flushing, tingling, itching, and redness of the face, arms, and chest, as well as headaches, and these are documented to be transient. Other minor, transient, and occasional side effects include upset stomach, intestinal gas, and dizziness. Participants will be informed about these potential side effects, and they will be monitored throughout the drug administration visits.

1.3.3.3. Potential risks of changes or delay in participant treatments being received prior to screening

Participants will be asked to refrain from starting new medications, including OTC supplements, during the procedures involved in this study. Should circumstances warrant a change in treatment, then participants will be asked to immediately notify the study team. If medically warranted, the participant begins to experience a decline, or their provider believes a treatment change is necessary, then the participant will be withdrawn from the study and encouraged to seek treatment.

For participants who are tapering and discontinuing antidepressant medications prior to entering the study, the following safety measures will be taken:

- Participants will be required to discuss changes in their antidepressant medication with their own prescribing provider, and to provide the study staff with a written note from the provider describing the plan.
- Study staff will be in contact with a participant who is tapering their antidepressant medication weekly with a phone safety assessment that will involve use of the C-SSRS. (These safety assessments are not designated as a ‘study visit’ so do not appear in the study visit flowsheets in Section 3.1 that summarize research procedures).
- Participants will be required to provide the name of at least one adult who will be in continuous contact with them during the taper and discontinuation period.

1.3.3.4. Risk of Emotional Responses, Discomforts During Administration & follow-up meetings.

During preparation, medication dosing, and integration sessions, participants will be asked to think about and to discuss their thoughts and emotions about their experiences working in the COVID pandemic, including the impact that these experiences have had on their lives. They may experience emotional responses to speaking about these thoughts, feelings, and concerns. The medication dosing session will take place at Harborview Medical Center and will be staffed by two investigators. The PI and/or investigators who will be present during the drug session will meet the patient at least 3 times prior to the medication dosing session. This will be done as part of psychoeducation that provides the participant with expectations for what to expect during the sessions.

1.3.3.5. Risks from routine medical evaluations.

The other risks involved in this research are generally considered minimal and include those normally associated with clinical care or research such as psychiatric evaluations, medical examinations, lab work - urine drug screening, blood draws, and research questionnaires.

Psychiatric Evaluations. Participants must answer personal questions, which may feel unpleasant or tiring, or may find discussing their symptoms andtheir illness during the psychiatric evaluation unpleasant or upsetting. Screening is necessary to assess participant eligibility. The major disadvantages of these assessments are the time that it takes to complete them. These clinical and research methods represent minimum risk(s).

Blood draws and urine tests for routine screening, including pregnancy testing. Participants will undergo a blood draw for a comprehensive metabolic panel, pregnancy test, liver panel. These tests prior to the psilocybin session allow the investigators to ensure that there are no contraindications to subjects' participation in the study, such as liver function abnormalities which could produce higher than expected levels of psilocybin. Higher levels of psilocybin may produce prolonged duration or greater intensity of subjective effects. Weighing the risks and benefits of a blood draw versus potentially unpredictable levels of psilocybin, the liver panel conveys more benefits than risks. Serum pregnancy and / or urinary pregnancy testing will be conducted at the screening visit to avoid the risk of unintended psilocybin exposure in utero. Less than 50mL will be drawn per collection. There will be a single blood draw over the course of study. The blood draw may produce temporary discomfort as a result of sampling blood, and discomfort or bruising at the blood drawing site. The urinary drug screen is necessary to verify abstinence from illicit substances or nonprescription stimulants prior to the psilocybin session.

1.3.3.6. Reproductive Risks

While some women who choose to participate in the study will be post-menopausal, so the pregnancy will not be possible, pre-menopausal women who could bear children could be at reproductive risk. There have been no systematic studies of the effect of psilocybin in utero, but also no case reports of birth defects resulting from psilocybin use. Thus, the reproductive risks seem minimal, but are not well characterized. We will screen all participants who are pre-menopausal women with pregnancy tests, and counsel them to use birth control until after their medication session.

1.3.3.7. Risks of privacy and confidentiality

There is a special potential risk to study participants because of their profession. Doctors in some states are required to report any mental health condition that may affect their performance to the state licensing board. If not mitigated, these risks could lead to reputational damage and even damage to their employability. The same requirement is not mandated for advance practice providers or nurses, but those clinicians could still face reputational damage if their participation in this study were made public. To mitigate this risk, we have applied for an FDA Certificate of Confidentiality, and all data will be entered using a study code.

1.3.3.8. Minimizing participant risks

The anticipated risks and risk mitigation strategies that will be used are summarized in the Table below.

Anticipated risk	Risk mitigation strategies	Occurrence of risk in recent published studies since 2000
Changes to any treatments a participant is receiving prior to study screening and enrollment	1. Clear communication with potential participants about existing treatments (for example need to taper SSRI before study entry).<br>2. Coordination between PI and participants mental health provider.	Published studies do not report numbers of participants who had complications related to changing treatments prior to screening.
Worsening of participant mental health symptoms during the study	1. Weekly phone calls from study staff to check in re participant well-being are part of the study procedures.<br>2. Mental health symptoms will be assessed by study therapists during all preparation and integration psychotherapy sessions, and therapists will discuss with participants and the study team (at a weekly meeting or urgently with the PI) any issues that may warrant a change in study procedures.	This risk has not been reported in published studies since 2000.
Increase in participant suicidality during the study	1. All participants will be screened with the Columbia Suicidality Screening instrument at preparation, medication days, integration sessions, and at follow up measurements.<br>2. If an increase in suicidality is measured, study staff will notify the PI immediately and arrangements for appropriate evaluation will be made with the attending psychiatrist on call at Harborview Medical Center.	This risk has not been reported in published studies.
Intense psychological reactions during the medication session	1. All study therapists will have training specifically for this issue.<br>2. the therapist manual lays out first-line and second-line interventions (which are non-pharmacologic), as well as indications about when to notify the PI Dr Back. These interventions are based on consultation and experience at other academic centers where research with synthesized psilocybin has been conducted (NYU, Yale, Johns Hopkins).	This risk has been reported in up to 40% of participants. In all instances, these reactions have been managed with non-pharmacologic interventions, and

		the reactions have been transient.
Discomfort resulting from study questionnaires	1. All participants will be informed that they may take a break at any time from questionnaires and that study staff are available to discuss issues that warrant follow up.	This risk has not been reported.
Confidentiality of participant identity	1. Because doctors in some states are required to report any mental health condition that may affect their performance to the state licensing board, we have submitted a request to the FDA for a Certificate of Confidentiality.	No prior studies have had participant inclusion criteria that specify doctors and nurses.
Reproductive risks	1. All female potential participants will be asked about their menopausal status at screening, and pre-menopausal participants will undergo a pregnancy test.	As noted above, the reproductive risks seem minimal, although they are not well characterized.
Length of study for subjects who were taking medications prior to study entry and who are randomized to placebo	1. The study team will clearly communicate with potential subjects during consent conversations to ensure eligible subjects know they may be off their medications longer if they are assigned the placebo group and then chose the open-label psilocybin session following un-blinding. We will recommend that individuals taking medications that need to be stopped, discuss this with their provider(s), family, and any other trusted people, prior to agreeing to participate in the study.<br><br>2. Minimize the time for subjects who are randomized to the placebo group to an open-label psilocybin session. We anticipate that arranging and scheduling an open-label psilocybin session will take 1-2 weeks. During this time, subjects will be followed weekly and will have an additional preparation psychotherapy session, and every weekly contact will include administration of the C-SSRS suicidality screen to minimize risk to these subjects. We judge that this additional risk of 1-2 weeks off antidepressants is acceptable given	This risk has not been reported.

	that these subjects will be monitored closely.	

A detailed therapist manual outlines procedures for every contact session with participants, and for participant issues that could represent risk, the manual outlines responses to minor, moderate, and severe side effects with first-line, second-line, and indications to call the PI by phone for urgent or emergent issues. Research staff including the investigators and PI will be available to evaluate any change in a participant's condition. Participants will be asked to contact us at any time if any unpleasant effects occur and will be contacted by study staff at least weekly during study participation. A study physician will be at the study site on medication dosing days to manage psychiatric emergencies.

Synthesized psilocybin: Reviews of the drug and the literature indicate that the physiological and psychological effects are transient. The patient will be monitored at all times by trained research and/or medical staff. The study medical doctors and nursing staff who are trained in dealing with drug challenge studies (such as ketamine, psilocybin, yohimbine, and various cannabis compounds) will remain in attendance during the entire session. Participants will be asked to remain on the CNRU until effects wear off. Procedures will be immediately stopped if participants request or if they are exhibiting significant distress. To minimize risk associated with synthesized psilocybin, participants will undergo a thorough screening for contraindicated psychological conditions or interacting medications. Dosing with psilocybin must be performed in a setting that minimizes distraction and interruption, and the participant must be attended following the dose by research staff trained in providing reassurance and a safe environment until the effects of the single dose have dissipated.

The PI may prescribe a designated rescue medication in the event of symptoms that require it during or after the medication dosing session. Rescue medications may be a benzodiazepine, zolpidem, or other anxiolytic or sedative according to the physician's clinical judgment.

Following the investigator brochure, although there have been no reports of their use in well-reported clinical trials with oral psilocybin, medications will be available for the treatment of causal symptomatic hypertension, agitation, or severe psychosis. Typically, these supplies are two dosage unitsof labetalol, nitroglycerin, lorazepam and/or diazepam, and risperidone or similar orally- disintegrating antipsychotic.

If any participant presents with an adverse reaction to the psychopharmacological agent as reported by them or assessed by a member of the research team, we will discontinue the study and call for medical assistance. Finally, the participant may be withdrawn from the study if his/her withdrawal is in the best interests of either the participant or of the research (e.g., due to a medical condition making the procedures unsafe or any condition making interpretation of the results difficult).

Niacin: (active-placebo-control agent): There is no reason to believe that niacin would exacerbate symptoms of depression or burnout or PTSD. Participants will be informed about Niacin's potential side effects, and they will be monitored throughout the drug administration visits. The rationale for using an active-placebo-control agent rather than active low dose of psilocybin (ranging 2mg -4mg), which have been used in the recent palliative care studies is because a partial response has been reported even with these low doses.

Changes or delays in treatments being received prior to screening: Participants will be asked to refrain from starting new medications to allow the participant and this study to safely proceed. If medically warranted, or the participant begins to experience a decline, or their provider believes a treatment change is necessary, then the participant will be withdrawn from the study and encouraged to seek treatment.

Involuntary holding, overnight for safety: The medical staff will closely monitor the participant's medical status during study administration. The staff take whatever medical steps are safely possible to reduce the need for an additional holding period including ongoing evaluations during the testing period. However, if there was concern about participants being a risk to themselves or others during the study, or if investigator feels it is medically necessary, and/or if the participant is not compliant, then the participant may need to be involuntarily held for his/her own safety. If a participant requires overnight observation, the participant's medication would be unblinded to maximize the information available to the treating inpatient team. Participants will be discharged after the study physician believes that it is safe to do so.

Emotional Responses and Discomforts During Psilocybin Medication Session, and follow-up appointments: Trained staff will be on hand to help participants deal with their responses to the study. The therapist manual outlines first- and second-line interventions for emotional responses or any psychological reactions that may be experienced. In studies since 2000 conducted at academic research centers, no participants required 'rescue' medication for psychological reactions—they were all managed with non-pharmacologic interventions.

Administration and follow-up appointments: Only the trained clinical staff will conduct such sessions. Staff will assist patients who require help with their anxiety. The anxiety experienced is no more than normally reported in any standard therapeutic session.

Psychiatric evaluation and clinical assessments: All participants must first give permission for the investigators to review medical records and later undergo a psychiatric evaluation and a urinary drug screen to ensure that participants are eligible to be in the study. We will try to schedule and to complete tasks quickly. Each participant is told that he/she does not have to answer any question that he/she does not want to answer, but that the questions about suicidal ideation and behaviors, including the complete C-SSRS, are required to participate in this study. Only the trained clinical will conduct these evaluations and all exercise utmost sensitivity.

Routine labs: Screening is necessary to assess participant eligibility and to uncover sometimes unknown contraindications. We minimize risk by having only skilled medical staff conduct the brief physical examination rule out any cardiac abnormalities.

Urine toxicology and blood draws. We minimize medical risk by having skilled nurses and phlebotomists do our blood drawing. If a blood draw produces temporary discomfort, then nursing staff can offer ice or cool compresses. There is also a remote possibility of inflammation or infection at theblood drawing site. Liver panel results will allow the investigators to detect poor metabolism, which could produce higher than expected levels of psilocybin. Higher levels of psilocybin may produce prolonged duration or greater intensity of subjective effects. Weighing the risks and benefits of a blood draw versus potentially unpredictable levels of psilocybin, the liver panel conveys more benefits than risks. The urinary drug screen is necessary for verifying the participant's report of current substance use and to eliminate potential risks of interactions of the study medications with other medications needed by the participant. Subjects are informed of this testing in advance of

the screening appointment and may choose to not participate in the study.

Study questionnaires. The research staff will inform all participants that they may take breaks while completing questionnaires, or stop if they are experiencing too much frustration, or anxiety. Participants may take breaks from completing measures and online tasks. Ratings will be administered with sensitivity by trained staff, either on paper instruments or online portals utilizing secure servers and data capture systems. All online responses will be captured on secure servers. This use of online measures speeds up the assessment process and seems to reduce some subject stress. All information will be kept strictly confidential.

Returning to work. Participants will be required to wait 48 hours after a medication dosing session before returning to work.

1.3.4. Confidentiality

All participant records, paper or electronic, will be stored in locked offices and password-protected encrypted drives that will be accessible only to the principal investigator and other study personnel. Research databases will be coded (all identifiers except id number will be removed) during data analyses and are also kept on secure servers or encrypted computers.

We have requested from the FDA a Certificate of Confidentiality for this study to protect the identities of participants.

2. Study Design and Endpoints

2.1 Description of the Study Design

This is a randomized, double-blind, placebo-controlled study that offers participants randomized to the placebo arm the option to receive open-label psilocybin after primary outcome measurements have been completed.

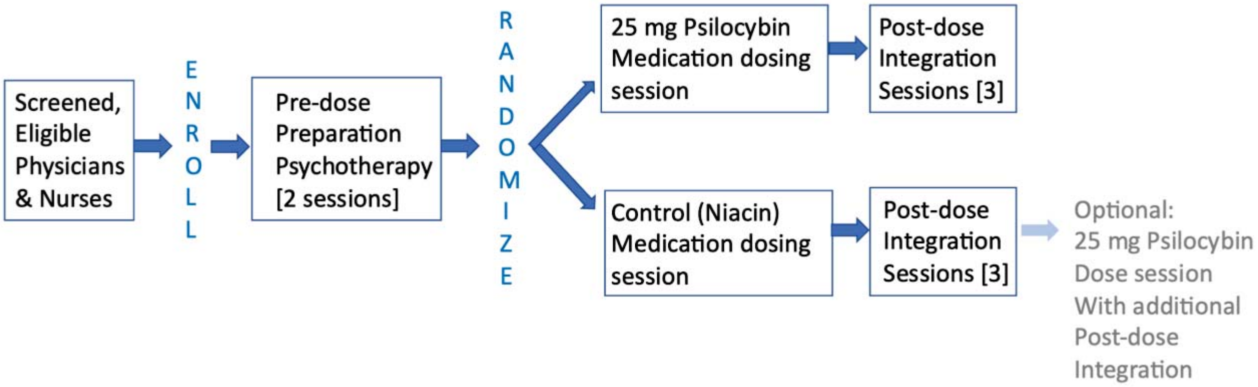

2.2. Research Methods and Design

This study tests a hypothesis that a single session of psilocybin (the 'medication dosing' session) in the context of pre- and post-dose psychotherapy will result in improvement of symptoms of depression and burnout measured 4 weeks post-dose. This study hypothesis will be tested in a single site, double-blind, randomized controlled design involving 30 clinician participants that will compare effects of a single 25mg oral dose of psilocybin to a 100 mg of niacin (active placebo). The primary outcome measurements will be collected 4 weeks after

the psilocybin dose, after which the participant group assignment will be unblinded, and participants who received niacin will be offered the opportunity to have a second dose session with a 25 mg dose (with pre- and post-dose psychotherapy).

Precautions will be taken to reduce potential for COVID exposure, following FDA guidance (https://www.fda.gov/media/136238/download):

- Some psychotherapy sessions can be conducted by video, although at least one preparation session and one integration session will be done in person.
- COVID-19 screening procedures mandated by UW Medicine will be followed per UW Medicine policy. Currently UW Medicine policies require a basic symptom check before ambulatory care encounters, for example.
- Participants will be tested for COVID using a rapid antigen test on the morning of their medication session, and therapists will also be tested on the morning of the participant's medication session.
- Physical distancing will be maintained as much as possible.

2.3. Study Endpoints

2.3.1. Primary Endpoint

The primary endpoint will assess symptoms of depression using the Montgomery-Asberg Depression Rating Scale (MADRS), a clinician-assessed instrument, at 4 weeks post psilocybin-assisted psychotherapy vs control psychotherapy. We expect at least 50% of participants treated with psilocybin to have a 35% reduction in their MADRS compared to baseline, which is a clinically significant difference compared to the control arm.

2.3.2. Secondary Endpoints

- Assess symptoms of depression using the Montgomery-Asberg Depression Rating Scale (MADRS) prior to the primary endpoint at 1 week post psilocybin-assisted psychotherapy vs control psychotherapy.
- Assess symptoms of depression using the MADRS after psilocybin administration (for patients randomized to psilocybin or who receive open-label psilocybin) at 1 week, 4 weeks, 8 weeks, 12 weeks, and 6 months.
- Assess symptoms of burnout using the Stanford Fulfillment Index (SFI) at 4 weeks post medication administration. We expect at least 50% of participants treated with psilocybin to show a reduction in the SFI work exhaustion and interpersonal disengagement subscales by at least 25% (from "a lot" to "moderately").
- Assess symptoms of burnout after psilocybin administration (for patients randomized to psilocybin or who receive open-label psilocybin) at 1 week, 4 weeks, 8 weeks, 12 weeks, and 6 months.
- Another secondary endpoint will assess symptoms of post-traumatic stress using the PCL5, measured at 1 week and 4 weeks after the medication session. We expect at least 50% of participants treated with psilocybin to show a reduction in PCL5 scores by at least 10 points (the clinically meaningful threshold for improvement).
- Assess symptoms of post-traumatic stress using the PCL5 after psilocybin administration (for patients randomized to psilocybin or who receive open-label psilocybin) at 1 week, 4 weeks, 8 weeks, 12 weeks, and 6 months.
- A 10% or less difference in the rate of adverse events between the treatment and control arms at all study visits.

2.3.3. Exploratory Endpoints

To test hypotheses concerning psychological therapeutic mechanisms, we will assess <u>correlation</u> and <u>mediational</u> influence of self-reported mystical states and psychological flexibility on changes in primary and secondary outcomes.

Exploratory endpoint A: Intensity of mystical experience (measured at the end of the medication dosing session), using the validated Mystical Experience Questionnaire. The hypothesis is that this will be a significant mediator of improvements in symptoms of depression and burnout.

Exploratory endpoint B: Psychological flexibility, using the AAQII, measured at 4 weeks after the medication dosing session. The hypothesis is that this will serve as a mediator of burnout.

Exploratory endpoint C: Interoceptive awareness, using the Multidimensional Assessment of Interoceptive Awareness.[48] The hypothesis is that this will be a mediator of improvements in symptoms of burnout.

3. Research Materials and Measures

3.1. Research Procedures

Participants will complete self-report and clinician-administered measures at baseline, prior to the medication dosing session, and at designated study visits (see figure next page).

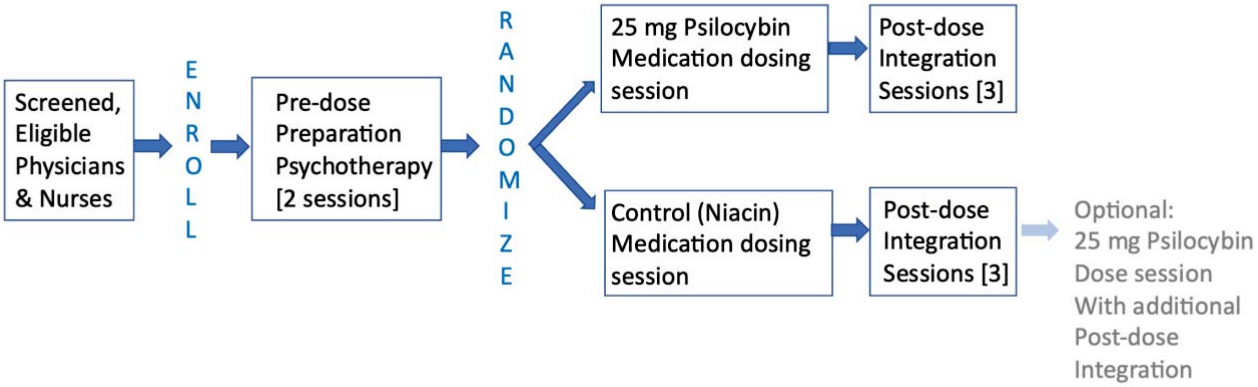

Schedule for Activities during Randomized Phase								
**Purpose**	**Screen**	**Prep**	**Prep**	**Medi-cation**	**Inte-gration**	**Inte-gration**	**Inte-gration**	**Follow-up**
**Study day (approx)**	**-21-14**	**-7**	**-1**	**0**	**1**	**8**	**15**	**28**
**Visit number**		**V1**	**V2**	**V3**	**V4**	**V5**	**V6**	**V7**
**Research activity**								
Demographics	X							
Eligibility Determination	X							
Medical exam	X							
Informed consent	X							
Caregiver contact info	X							
Blood draw	X							
Pregnancy screen (if applicable)	X			**X**				
PI review of inclusion/exclusion	X							

Informed Consent		**X**						
Mental health evaluation		X						
Education re expectations	X	X						
Preparation psychotherapy		X						
Urine screen	X							
Randomization				X				
Medication (psilocybin or control)				X				
Medication dosing monitoring				X				
Integration psychotherapy					X	X	X	
Adverse event reporting				X	X	X	X	
Un-blinding								X**
**Measures**								
Mont-Asberg Depression (MADRS)	X	X			X	X		X
Beck Depression Scale		X			X	X		X
Stanford Fulfillment Index		X			X	X		X
Post-traumatic stress (PCL5)		X			X	X		X
Likelihood to leave job		X			X	X		X
Self-reported mystical exp				X				
Moral injury symptom scale		X						X
Psychedelic Music Questionnaire					X			
Psychological flexibility (AAQII)		X				X		X
Interoceptive awareness (MAIA2)		X			X	X		X
Suicidality (C-SSRS)	X	X	X	X	X	X	X	X
Qualitative interview								X
Adverse events, Suicidality	X	X	X	X	X	X	X	X

**The unblinding will be done after the study coordinator has verified that the Day 28 measures have been completed.

**Follow up measures for participants randomized to psilocybin**			
**Purpose**	**Follow -up**	**Follow -up**	**Follow -up**
**Study day (approx.)**	56	84	180
**Visit number**	V8	V9	V10
**Measures**			
Mont-Asberg Depression (MADRS)	X	X	X
Beck Depression Scale	X	X	X
Stanford Fulfillment Index	X	X	X
Post-traumatic stress (PCL5)	X	X	X
Likelihood to leave job	X	X	X
Psychological flexibility (AAQII)	X	X	X
Interoceptive awareness (MAIA2)	X	X	X
Suicidality (C-SSRS)	X	X	X
Qualitative interview			
Adverse events	X	X	X

3.1.1. Clinician administered measures

1. Montgomery Asberg Depression (MADRS)[49]

2. Columbia Suicide Severity Rating Scale (C-SSRS)[50]
3. Qualitative interview post-psilocybin

3.1.2. Self-report measures
1. Beck Depression Scale II[51]
2. Stanford Fulfillment Index (Burnout) (baseline and 4 weeks only)[52]
3. Post-traumatic stress symptoms (PCL5)[53]
4. Professional likelihood of leaving[10]
5. Self-reported mystical experience (MEQ30) (day of journey only)[54]
6. Moral injury symptom scale-Health Professionals Version[54b]
7. Psychological Flexibility (AAQII)[55]
8. Music experience questionnaire (day of journey only) [Mendel Kaelen, personal communication]
9. Multidimensional Assessment of Interoceptive Awareness (MAIA2)[48]

3.1.3. Initial Research procedures:

Phone Pre-screen. Because of a large number of interested potential participants, a pre-screening procedure has been developed to reduce burden on potential participants by performing more detailed pre-screening prior to an in-person screening visit (this will allow people likely to be ineligible because of low symptom scores to avoid the in-person screening visit). During a phone pre-screening call, the potential participant will be sent a link to a version of the MADRS that has been adapted for self-administration (MADRS-S). The MADRS-S is designed for self-assessment, and correlates reasonably well with the clinician-administered MADRS that is being used as an eligibility criterion.(G. Bondolfi, F. Jermann, B. Weber Rouget, M. Gex-Fabry, A. McQuillan, A. Dupont-Willemin, J.-M. Aubry, C. Nguyen, Self- and clinician-rated Montgomery–Åsberg Depression Rating Scale: Evaluation in clinical practice, Journal of Affective Disorders, Volume 121, Issue 3, 2010, p 268-272). The online pre-screen MADRS-S will not collect any identifying information and will calculate a total score, which will be available to the study coordinator at the time of the phone call. The study coordinator will inform the caller whether their score is in a range (19 or above) that they will likely meet the eligibility criteria for the study . The pre-screen MADRS-S score will only be used for pre-screen phone calls and will not be used as a study measurement.

In-person screening visit. At the study screening visit, the study, the conditions, and the procedures involved, will be explained, and discussed with each subject. Each participant will have a chance to ask questions and to receive answers before making a final decision whether to participate. Participants who decide to participate will document their informed consent. For safety, after completing the consent, the participants may designate 2 adults who will be expected to have continuing contact with the subject, who may provide patient transportation, and who may observe changes in the participants' behavior and attitude that warrant notification of the research staff and/or PI. The screening visit will include the clinician-administered MADRS, and to be eligible for the study a MADRS score of 20 or greater is required.

3.1.4. Study Research Evaluations and Procedures

Medication and lifestyle changes may be required of all participants. All participants will be required to taper off and discontinue use of antidepressants (such as SSRIs) before starting the study and cannot take antidepressants during the study even if they were taking them before study entry. Participants who were taking antidepressants before

study entry will need to be off them for five half- lives of the drug plus 2 weeks before starting the study and will be required to stay off their medication until after they have completed the primary outcome measurements on day 28. After unblinding (as early as day 28, after primary outcome measurements are received). Subjects who were randomized to psilocybin will be able to resume antidepressants in consultation with their provider. Subjects who were randomized to placebo will need to stay off their antidepressants if they wish to have an open-label psilocybin medication session, and after their open-label psilocybin session they will need to stay off their medication until at least day 29 post-open-label-psilocybin, until after they have completed the day 28 post-open-label-psilocybin outcome measurements. If a participant randomized to placebo does not wish to have an open-label psilocybin medication session, they will be able to resume their antidepressants after their primary outcome measurements in consultation with their provider, as early as day 29 post-open-label-psilocybin. They will also need to abstain from tobacco products, alcohol, or any recreational drugs such as marijuana during the study. Study personnel may obtain a release to contact the participant's prescribing provider to discuss current prescriptions and medication history. This is decided on a case by case basis upon the discretion of the PI.

Medication contraindications will include anti-seizure medications, insulin and oral hypoglycemics, hypertension medications that act on the central nervous system (clonidine and aldomet specifically), cardiovascular medications and the following psychotropic medications: anti-psychotics (first and second generation agents), anti-depressants and mood stabilizers. Psychotropic medications cannot be taken in the previous two weeks, with Prozac (fluoxetine) not being taken within the last 5 weeks prior to receiving any study agents. Participants may only take prn (as needed) benzodiazepines (i.e., lorazepam) up to three days before the session. Participants will refrain from taking any medications the day of either the experimental psilocybin medication dosing or active-placebo-control medication dosing session, except prescribed opiate pain medication or over-the-counter non-narcotic pain medication at any time. It will also be necessary to refrain from drinking alcoholic beverages the day before, the day of, and the day after each medication dosing session.

Physical exam, and blood and urine tests are required at the screening visit to meet eligibility criteria. As part of the study participants will have a physical examination, and screening blood (including a comprehensive metabolic panel, chemistries, hematological tests) and screening urine toxicology tests (for medications as described in Section 1.3.3.8; and pregnancy for female participants of childbearing potential). These results will also become part of the research case record.

Randomization and One-time Administration of the Study Medications Psilocybin or active- placebo-control Niacin. Psilocybin and niacin (active-placebo-control agent) will be administered in identically appearing opaque capsules with approximately 180ml of water. The dose of psilocybin will be 25 mg, which has been recently demonstrated to be as effective as a body weight-adjusted dose.[56] The dose of niacin will be 100 mg, and GMP niacin will be provided by the Usona Institute. In the study, half of the participants will be randomized to receive psilocybin (n=15) or the active-placebo-control, niacin (n=15). Following the first treatment session of either the active agent or active-placebo- control, participants who were randomized to receive active-placebo-control will be offered the option to receive open-label psilocybin. (The blind will be broken after Day 28 to make this determination; this is after our primary endpoint which is collected on Day 28. The study rater (for clinician-administered measures) wlremain blinded to the treatment assignment. Data will be collected from all subjects after open-label treatment for descriptive purposes, but these data will not be analyzed as part of our primary analysis plan.

Clinical Assessments and Study Evaluations. Online surveys and measures will be offered using secure data capture systems, using online or paper documents. These evaluations will include self-report questionnaires and clinician administered measures. This testing may last up to 1 to 2.5 hours.

Research study measures, follow-ups and assessments are outlined (in Tables below) and represent study specific clinician administered and self-administered measures. Safety measures and checks will be completed at some study visits: vital signs, adverse events, spontaneously reported reactions, general well-being, medications, and changes to any medications.

All participant responses to measures and assessments will be recorded either on traditional paper formats and scanned to a secure server, or in online data capturing systems which instantly upload responses or surveys to a secure server. All portable digital participant data will be uploaded immediately and stored in secure databases, on password-protected computers in locked offices, and on secure servers.

3.1.5. Study Environment

Research visits and the medication dosing session will be conducted at Harborview Medical Center. The screening, consent, preparation, and integration visits will occur at the Virology Research Clinic at Harborview Medical Center in the Ninth and Jefferson Building on the 11th floor. These visits will occur in a clinic room designed for outpatient, non-procedural visits, and this is a private room that is equipped with comfortable chairs and a window, appropriate for psychotherapy visits. This clinic is dedicated research space that is a non-CRBB space and is directed by Dr. Anna Wald.

The medication sessions will occur at Harborview Medical Center, in the Sleep Lab. These research visits will occur during the day when the clinic is not in session, in a room used for nighttime sleep studies. These are private, quiet rooms that are outfitted like hotel rooms with minimal equipment, a comfortable bed, art prints on the walls, chairs for therapists, and a private bathroom.

3.1.6. Study Enrollment and Withdrawal

Participant Population – physicians and nurses with moderately severe symptoms of depression. Participants meeting study inclusion / exclusion criteria after careful screening and examination will be invited to participate and will be provided with all the information needed to provide an informed consent. We will recruit 30 adult participants (25 to 65 years) of both sexes with clinically significant symptoms of depression.

4. Participant Inclusion and Exclusion Criteria

4.1. Inclusion Criteria

a. Participants must be physicians or nurses or advance practice providers (nurse practitioners or physician assistants) with at least 1 month of frontline clinical experience during the COVID pandemic who rate at least 2 of 4 items from the COVID Exposure index as ‘more than half the days’ during their peak 2 week period of exposure:
   - i. Caring for someone critically ill with COVID-19, or who became critically ill while you were involved.
   - ii. Working longer hours than usual in order to provide assistance or care to individuals with COVID-19
   - iii. Witnessing or responding to a death related to COVID-19, or losing a patient you had been caring for to COVID-19
   - iv. Caring for patients who have died without family physically present due to COVID-19 precautions

b. Have a Montgomery-Asberg clinician-administered depression score ≥21, indicating moderately severe symptoms. This measurement is clinician-administered and will be performed by a study team member who is not a therapist, who will receive special training.
c. Have had persistent symptoms despite at least one medication and/or therapy trial of standard care treatment for depression.
d. English speaking – able to understand the process of consent and the risk and benefits associated with the study, and able to give written informed consent.
e. Must be willing to sign a medical release for the investigators to communicate directly with their therapist and doctors to confirm a medication and/or medical history. This is decided on a case by case basisupon the discretion of the PI.
f. Must be driven home after the medication dosing session by a driver(which could be a friend, family, rideshare or taxi).
g. Must provide at least one adult to have continuous contact with the participant, provide participant transportation, monitor changes in the participant's behavior, and notify research staff of behavior changes.
h. Has been off selective serotonin inhibitors for five half-lives of the drug plus 2 weeks.
i. Must avoid taking any psychiatric medications or starting a new psychiatric medication during the study. Should participant's doctor recommend starting a new psychiatric medication, participant will be required to notify the study team and the subject would withdraw from the study
j. Must provide a contact (relative, spouse, close friend, or other caregiver) who is willing and able to be reached by the Clinical Investigators in the event of a participant becoming suicidal.
k. Must have a negative pregnancy test at study entry and prior to eachmedication dosing session if able to bear children and must agree to use adequate birth control.
l. Are willing to commit to preparation sessions, medication dosing sessions, integration sessions, to complete evaluation instruments and commit to be contacted for all necessary telephone contacts.

4.2. Exclusion Criteria.

a. Personal or immediate family history of schizophrenia, bipolar affective disorder, delusion disorder, paranoid disorder, or schizoaffective disorder.
b. Suicidal ideation with a C-SSRS ≥ 3
c. Current substance abuse disorder (except in the case of mild alcohol use)
d. Neuroleptic and SSRI medications that cannot be tapered and discontinued in conjunction with the participant's prescribing physician.
e. Unstable neurological or medical condition; history of seizure, chronic/severe headaches.
f. Positive urine pregnancy test at the time of screening
g. Any unstable medical condition that my render study procedures unsafe based on the medical history performed as part of the screening procedures, including active cardiac medical issues such as ongoing angina, uncontrolled hypertension, or recent cardiac ischemia requiring medical attention (within the past 3 months
h. Any use of psychedelic drugs within the prior 12 months.
i. Use of tramadol, due to the potential for serotonin syndrome with concomitant use of

psilocybin.

j. Allergy to niacin (N.B. the investigators will inform participants that flushing, redness, tingling, and headache are normal side effects that do not predict anaphylaxis, which although reported is extremely rare).

k. Individuals who are on MOAI (monoamine oxidase inhibitors) or who have a known sensitivity to the drug or its metabolites. Psilocybin is contraindicated in medications that are known UGT (UDP-glucuronosyltransferase) enzymemodulators. The concurrent use of SSRI/SNRI meds is assumed to be contraindicated due to the potential to increase the risk of serotonin syndrome and/or to attenuate the binding of psilocin to the HT2A receptor.

4.3. Medication management

Medication contraindications will include anti-seizure medications, insulin and oral hypoglycemics, certain hypertension medications (clonidine, and aldomet specifically), cardiovascular medications and the following psychotropic medications: anti-psychotics (first and second generation agents), anti-depressants and mood stabilizers. Psychotropic medications cannot be taken in the previous two weeks, with Prozac (fluoxetine) not being taken within the last 5 weeks prior to receiving any study agents. Participants may only take prn (as needed) benzodiazepines (i.e., lorazepam) up to three days before the session.

Participants will refrain from taking any medications the day of medication dosing session, except prescribed opiate pain medication or over-the-counter non-narcotic pain medication at any time. It will also be necessary to refrain from alcohol the day before, the day of, and the day after each medication dosing session.

Participants must agree to:

a. Ingest only alcohol-free liquids after 24:00 (midnight) the evening before the Medication Dosing Session

b. Refrain from the use of any exclusionary medications. Except in the case that the PI prescribes benzodiazepine or zolpidem for insomnia.

c. Refrain from use caffeine or nicotine for two hours before and six hours after ingesting the drug, or until research staff deem it safe to do so.

4.4. Strategies for Recruitment and Retention

Participants will be recruited through social media and professional organizations (i.e., Northwest Healthcare Response Network—the organization that has coordinated the clinician response to the COVID pandemic in Washington state). Because eligible participants will be physicians and nurses, we anticipate a general familiarity with clinical trials, although not for a trial involving PAP. An informational website will be created to enable potential participants to understand the study purpose, inclusion criteria, exclusion criteria, and study email and phone number.

4.5. Participant Withdrawal or Termination

Participants may withdraw their consent for use of their data and/or terminate participation in the study at any moment during the study by calling the Principal Investigator or a member of the research team. To revoke authorization of the use and disclosure of Personal Health Information, the participant must follow-up the phone call by submitting a request in writing to the Principal Investigator. In this event, the Principal Investigator will indicate in the data base that consent from the participant is no longer active, and that data can no longer be used.

A participant may be withdrawn from the research for any of the following reasons:

(1) A participant will be withdrawn if they become actively suicidal
(2) A participant will be withdrawn if during the preparation visits, they show signs of severe personality disorder that was not evident in the screening visit.
(3) A participant may be withdrawn if their withdrawal is in the best interests of either the participant or of the research (e.g., due to a medical condition making the procedures unsafe or any condition making interpretation of the results difficult).
(4) A participant may be withdrawn if they are unable to comply with study visits (e.g., they miss their preparation visits without explanation).

4.6. Premature Termination or Suspension of Study

The Principal Investigator is responsible for monitoring the data, assuring protocol compliance, and conducting quarterly safety reviews. During the review process the principal investigator will evaluate whether the study should continue unchanged, require modification/amendment, or close to enrollment. Likewise, the principal investigator will periodically review the collection, storage, and distribution practices associated with clinical data bank, and determine whether changes to enhance confidentiality and privacy are required.

The Principal Investigator, the Medical Monitor and the IRB have the authority to stop or suspend the study or require modifications.

5. Study Agent

Synthesized psilocybin, the study drug, is a 4-hydroxy-N,N-dimethyltryptamine and occurs in nature in many species of mushrooms, including the genera Psilocybe, Conocybe, Gymnopilus, Panaeolus, and Strophparia. Its chemical formula is $C_{12}H_{17}N_2O_4P$. Psilocybin is a potent agonist at 5-HT2A/C, and their binding potency to these receptors correlates with human potency as hallucinogens [7]. Psilocybin has been administered to normal volunteers to measure physiological and psychological parameters at doses ranging from 0.045mg/kg to 0.43mg/kg [45; 51]. It has also been safely administered to patients with OCD at doses ranging from 0.025mg/kg to 0.3mg/kg.

After the FDA has approved the content of the protocol, the psilocybin will be provided by the Usona Institute, Inc., 2800 Woods Hollow Rd. Madison, WI 53711, who will provide this information as a supplement to this IND application. The Usona Institute utilizes a contract manufacturing organization (CMO) to manufacture GMP psilocybin, and the CMO is Almac Pharma Services Limited, Seagoe Industrial Estate, Portadown, Craigavon, BT63 SUA, United Kingdom. Using a 222 DEA form, the psilocybin will be shipped to Dr Back to be stored in DEA safe located in the Investigational Pharmacy at Harborview Medical Center. To safeguard the psilocybin, a special storage safe will be approved by DEA to store only the psilocybin. Designated staff will be the only personnel with access to the psilocybin. Both the active and placebo will be prepared by designated study staff by a compounding process. The UW Investigational Pharmacy (IDS) will store and track the accountability of the psilocybin. A log of the psilocybin accountability, compounding, and release to the study team will be kept by the designated IDS staff. This will provide strict safeguarding and accounting of the agent both for internal and external regulatory agencies. The sponsor-investigator will be the DEA Schedule 1 license holder and will dispense the drug directly to study participants.

6. Study Procedure and Schedule

This study will use a randomized, placebo-controlled, double-blind design to examine the clinical and neural effects of psilocybin-assisted psychotherapy with either synthesized psilocybin (25 mg) or placebo (niacin 100 mg), given along with non-drug preparatory and integrative psychotherapy sessions. The duration of the randomized study phase is from consent until two weeks after drug administration, which is approximately 6 weeks. The blind will be broken at 4 weeks after measures have been completed, and the participants who received placebo will be offered the option toreceive open-label psilocybin. In addition to the baseline assessments and medical evaluation, the study will consist of atleast two Preparation sessions with the study therapists, the medication dosing session (which will require at least 7 hours), and post-medication session Integration sessions, starting the following morning after the medication dosing session and continuing at 2 weeks, 3 weeks, and 4 weeks post-medication. These integration sessions may be conducted by secure videoconference.

6.1. Randomization Blinded Study Phase

Upon completion of the Preparation sessions, participants will be assigned to one of the two conditions for the Medication Dosing session: the experimental condition (synthesized psilocybin 25mg) or placebo (niacin). The randomization will be performed by IDS and capsules will be placed in envelopes for each participant. Randomization will be stratified by profession (physician vs nurse) and ethnicity (Black or Hispanic vs other).

Psilocybin and niacin will be administered in identically appearing opaque, size 0 gelatin capsule with approximately 180ml of water. The dose of psilocybin will be 25 mg and the niacin will be 100 mg. Each participant will have the option of participating in the medication dosing sessions. In the study, half of the participants will be randomized to receive psilocybin (n=15) or the placebo, niacin (n=15). Following the first treatment session of either the active agent or placebo, participants who were randomized to receive placebo will be offered the option to receive open-label psilocybin. (The blind will be broken at 4weeks to make this determination, after primary endpoint measures have been collected. Clinical raters will remain blind to the treatment assignment). Data will be collected from all subjects after open-label treatment for descriptive purposes, but these data will not be analyzed as part of our primary analysis plan.

The participant’s condition assignment will be maintained from randomization until the 4 week unblinding. If there is an adverse event or other emergency requiring knowledge of participant’s condition assignment, as when pharmacological intervention is necessary, the blind may be broken for an individual participant. In this case, the study raters who will administer the questionnaires will remain blind to the participant’s condition assignment.

Participants in the placebo condition will be eligible for an open-label study continuation. They will be re-screened after the blind is broken to make sure they still meet study criteria.If they are eligible and choose to enroll in the open-label continuation, they may do so, undergoing psilocybin-assisted sessions occurring within another seven to 14-day period.

The open-label phase of the study will follow nearly identical procedures to those used in the randomized phase. Participants will be re-assessed on all study measures following the same schedule of the randomized phase. Participants need not complete any portion of the open-label phase or the several follow up phases for their participation in the study to be considered completed.

6.2. Study schedule for participants by visit

6.2.1. Screening and Informed consent

Prior to participation in this research, subjects will receive a brief secure online screening survey and then if eligible, will be screened by phone to determine preliminary eligibility. All subjects will be informed about the phone screening and what the screening process involves.

All potential participants will be told that we collect demographic information, relevant medical history, and psychiatric history during the screening process. Screening data are entered directly into a secure online database and are considered a source document.

All seemingly eligible subjects will be told that they need to come in for baseline screening visit, and will be informed about baseline questionnaires, clinician assessment, blood draw, and pregnancy test (if applicable) to ensure their eligibility. Subjects are free to decline participation at this point or to discontinue participation at any future point and to withdraw from consideration.

Some potential participants live in another state, and it may not be feasible to have them come for an in-person screening visit. In this case we will conduct the screening interview using secure video communication platforms. We will ask these participants to send us their most recent physical, and blood work (recent as of 1 month from the screening visit will be allowed). This will be faxed to the PI and reviewed for potential participation. A remote urine test will be ordered for the screening process of remote participants.

Prior to obtaining informed consent, the PI will confirm that the participant meets all study inclusion and exclusion criteria. The investigator or research staff member obtaining consent will ask the participant to provide a brief summary of the study to ensure they understand what is being asked of them and any potential risks and benefits.

Note that if a female subject is pregnant, or breast-feeding, or does not agree to use an approved birth control method (i.e., oral, injectable, or implant birth control, condom, diaphragm with spermicide, intrauterine device, tubal ligation, abstinence, or partner with vasectomy) she will be excluded.

Findings of pregnancy, active cardiac medical issues, abnormal liver function, abnormal renal function, or abnormal blood counts, and any lab indicating an undiagnosed illness would exclude a participant from moving on to the preparation 1 visit. When screening requirements have been met, participants will have a visit with the PI and/or key study personnel in which the study, the scheduling and the procedures will be reviewed. All participants' questions will be answered prior to obtaining an informed consent.

After providing informed consent, the participant will complete initial measures. Note that randomization to the psilocybin or control (niacin) treatment groups will occur at the end of visit 2.

6.2.2. Preparation visits (Visits 1, 2)

Preparation for the medication dosing session (psilocybin or control) consists of 2 meetings for a total of 5-6 hours in the 2 weeks before the medication dosing session. The main purpose of the preparation meetings is to develop rapport and trust, which helps minimize the risk of fear or anxiety reactions during the psilocybin sessions. Additional meetings and contact hours will be scheduled if it is judged necessary to establish rapport and trust. Consistent with other published psilocybin protocols, the participant's life history and current situation in life will be reviewed, and intentions and expectations for the psilocybin sessions will be discussed. Audio or video recordings (with coded id number) of this session will be made to allow the participant and the investigator-therapists to review preparation sessions.

6.2.3. Medication Dosing session (Visit 3)

Procedures for psilocybin administration and the conduct of the session will be similar to procedures used in studies with psilocybin conducted at New York University, Johns Hopkins, and Yale. Participants will be instructed to consume a low-fat breakfast before reporting to the clinic room for the psilocybin sessions. Before psilocybin administration, urine pregnancy test will be conducted in females of childbearing potential, and a negative pregnancy test will be required in order to continue in the medication session. Participants and staff will also complete pre-session questionnaires. Before each psilocybin session, study personnel will interview the participant. If the investigator believes that the session is contraindicated, the session will be cancelled or postponed.

Psilocybin will be administered in opaque gelatin capsules with approximately 100 ml water. At least one therapist, under the supervision of the investigators, will be present in the room and available to respond to participants' physical and emotional needs during the full course of the session (at least 7 hours). A physician on the study team will be immediately available via pager or mobile phone for at least 3 hours or until the peak effects of psilocybin have subsided, whichever is longer.

During the session, participants will lie on a couch, wear eyeshades, and listen to a program of music through headphones. The participant will be encouraged to focus her or his attention inward. The eyeshades and music are intended to encourage this inward reflection. Heart rate and blood pressure will be measured pre drug administration, at 30, 60, 90, 120 minutes after drug administration, and then at least hourly until at least 6 hours after drug administration and until drug effects have subsided. Heart rate and blood pressure assessments will be obtained after the participant has been sitting or recumbent for at least 5 minutes. Sessions are expected to last approximately 7 to 10 hours. A sample schedule for the medication dosing session is as follows:

Approximate Time	***Procedure or Action***
09:00	Urine pregnancy test for females of childbearing potential, participant acclimates to environment, completes measures.
09:30	Baseline BP, Pulse, Temp.<br>**Medication Administration.**<br>Begin video recording.
10:00	BP, Pulse
10:30	BP, Pulse
11:00	BP, Pulse
11:30	BP, Pulse
12:30	BP, Pulse
13:30	BP, Pulse
14:30	BP, Pulse
15:30	BP, Pulse
16:30	BP Pulse.<br>Assessment for discharge home.

At about the same time of each heart rate/blood pressure measurement, monitors will complete notations in the Medication Day Case Report Form to rate the presence and intensity of behaviors, signs, and reported symptoms, including sleepiness, amount of speech, anxiety, stimulation/arousal,

tearing/crying,nausea/vomiting, yawning, restlessness, feelings of unreality, visual changes, euphoria, and peacefulness. Video and audio recordings will be made throughout the session, as allowed by participants.

Participants will be encouraged to lie comfortably in bed wearing eye shades during the first few hours of the experience and to listen to pre-selected music chosen by the study staff. Two team members will remain with the participants the entire time and beavailable for any processing of intrapsychic material made manifest by the treatment.

If participants experience or report on any adverse effects or if the research staff feelsthat at the end of testing, that the participant has any adverse effects, participants will be asked / required to remain until effects wear off and medically cleared by the PI. Medical assistance will be offered. The investigators will query participants about suicidal ideation or intent during screening, and prior to each psilocybin session. Participants who evidence suicidal ideation or intent will be withdrawn from the study and referred to appropriate psychiatric care.

In the event that participants experience distressing anxious symptoms during the drug session, the clinical personnel in attendance (the principal investigator and team member) will initiate verbal communication designed to reorient and reassure that participant. The occurrence and intensity of anxiety or panic responses can be reduced through providing participants with information on potential drug effects, supervision, and monitoring of participants for the duration of drug effects. The goal in this study is to treat participants undergoing anxiety or panic reactions first by verbal and psychological interventions and using anxiolytic medication only after verbal and psychological interventions have failed, and if participants are endangering themselves or others. Residual symptoms will be addressed during the frequent follow-up psychotherapy visits with the investigators.

At the end of the medication dosing session:

- o Participants will complete paper or computer-based questionnaires designed to assess acute subjective experiences associated with the medication dosing session.
- o Study therapists will the participant’s mood and suicidality with the C-SSRS.
- o Participants will also be asked to write a narrative description of the experience of the psilocybin session before their next in-person meeting.
- o Participants will remain under observation until both the participant and investigator agree that their perception, cognition, functioning, and judgment are no longer impaired by the medication.
- o Participants will be discharged in the company of the adult who is providing continuous contact.

6.2.4. Integration sessions (Visit 4, 5, and 6)

During the meetings with the study therapists after the medication dosing session, participants will complete questionnaire assessment measures. During visit 4, study therapists will also complete questionnaires retrospectively rating various behaviors and experiences observed on the session day and reported by the participant during the meeting (using the Next-Day Monitor Rating Form), and participants will be asked to discuss the narrative description of their medication session.

The purpose of the integration sessions is to ensure psychological stability after the medication dosing session and provide an opportunity for the participant to discuss thoughts or feelings from the session. These post-medication integration sessions allow time for reflection, meaning-making and psychological change. Audio or video recordings (with coded id number) of this session will be made.

6.2.5. Long-term follow-up

A study therapist who conducted the integration, medication dosing and preparation sessions will contact the participant at 3 months post-psilocybin, and 6 months post-psilocybin

(note that this takes into account participants who receive psilocybin after the primary endpoint measures have been collected in the open label phase of the study (see below). The therapist will have a 30 min phone call or video call with the participant, and the participants will fill out measures using a secure online portal.

6.2.6. Timing and Location of Meetings, Sessions, and Measures.

Although much effort is put in to scheduling participants within the stated time frames, these are only estimates. Variables out of the study team's control (e.g., participant availability, university closings, availability of study rooms, illness) may prevent the scheduling of volunteers within the stated time frame goals. There is no evidence to suggest that the difference in timing of sessions, meetings, and measures will adversely affect the volunteer or the validity of the study. On those occasions when the timing of meetings, sessions or measures deviate substantially from the time frames in the protocol, we will report these as deviations in the Continuing Review.

6.3. Open Label Psilocybin Sessions.

The participants who choose to receive open-label psilocybin will be re-screened after the blind is broken after primary endpoint measures are collected (week 4) to make sure they still meet study criteria. If they are eligible and choose to enroll in the open-label continuation, they may do so, undergoing a psilocybin-assisted session occurring within another seven to 14-day period.

The open-label phase of the study will follow nearly identical procedures to those used in the randomized phase except that a single preparation psychotherapy session will be given (see Schedule below, Study day -1). Participants will be re-assessed on all study measures following the same schedule of the randomized phase.

Participants need not complete any portion of the open-label phase or the several follow up support appointments for their participation in the study to be considered completed.

Data will be collected from all subjects after open-label treatment for descriptive purposes and will be viewed not as part of our primary analysis plan but as part of a secondary analysis examining the impact of PAP on symptoms of depression and burnout.

	Schedule for Activities during Open Label Psilocybin Phase									
**Purpose**	**Prep**	**Medi-cation**	**Inte-gration**	**Inte-gration**	**Inte-gation**	**Follow -up**	**Follow -up**	**Follow -up**	**Follow -up**	**Follow -up**
**Study day (‘denotes open-label)**	**-1’**	**0’**	**1’**	**7’**	**14’**	**21’**	**28’**	**56’**	**84’**	**180’**
**Visit number**	**V7**	**V8**	**V9**	**V10**	**V11**	**V12**	**V13***	**V14***	**V15***	**V16***
**Research activity**										
Psychiatric evaluation	X									
Education re expectations	X									
Preparation psychotherapy	X									
Urine screen		X								
Medication (psilocybin)		X								
Medication dosing monitoring		X								
Integration psychotherapy			X	X	X					
Adverse event reporting		X	X	X	X					
**Measures**										
Montgomery Asberg Depression	X		X	X	X	X	X	X	X	
Beck Depression Scale	X		X	X	X	X	X	X	X	
Stanford Fulfillment Index	X		X	X	X	X	X	X	X	
Post-traumatic stress (PCL5)	X		X	X	X	X	X	X	X	

Likelihood to leave job	X				X					
Self-reported mystical exp		X								
Moral injury symptom scale		X						X		
Psychedelic music questionnaire			X							
Psychological flexibility (AAQII)	X				X	X	X	X	X	
Suicidality (C-SSRS)	X	X	X	X	X	X	X	X	X	X
Qualitative interview						X				
Adverse events	X	X	X	X	X	X	X	X	X	

*Note that V13, V14, V15, and V16 are likely to be video or remote visits.

6.4. Rescue Medications, Treatments, and Procedures

The plan for managing psychiatric emergencies includes:

- A detailed therapist manual, and training for all study therapists on procedures in the manual for handling potential adverse events including agitation, hallucinations, anxiety, and other intense psychological reactions.
- The two-person therapist team that will be with the participant for the entire medication dosing session will include a licensed therapist or physician with training in psychiatric emergencies and adverse effects of psychedelics.
- A psychiatrist team member will be involved in screening and evaluation of all participants.
- A physician with training in psychiatric emergencies and adverse effects of psychedelics will be immediately available on site for the medication dosing days.
- A psychiatrist team member will be immediately available to assess any participant noted by study staff to have a significant change in mood or suicidality.
- In the event of a psychiatric emergency, the Harborview Medical Center Psychiatry Emergency Services in the next building is available 24 hours a day, with on-site psychiatrists and nurse practitioners.
- The PI may prescribe a designated rescue medication in the event of symptoms that require it during or after the medication dosing session. Study therapists who have performed first-line and second-line interventions (that do not involve medications) will have the ability to contact the PI by phone, who is available to assess patients within 20 min during every medication session. If a patient does have an adverse reaction that requires a rescue medication, participants will be monitored for up to 2 hours beyond the required 6 hour duration of the session. If at the end of that period that participant requires further, Dr Back (PI) will consult with the attending psychiatrist on call at Harborview Medical Center.

If participants experience or report on any adverse effects or if the research staff feels that at the end of testing, that the participant has any adverse effects, participants will be able to remain until effects wear off (likely only for a short period) and medical assistance will be offered. Participants will be free to withdraw from the study whenever they wish. In addition, if any participant presents with an adverse reaction to the psychopharmacological agent as reported by them or assessed by a member of the research team, we will discontinue the study and call for medical assistance

6.5. Safety Plan for Suicidality:

During all study visits a study investigator with clinical training will assess the participant's well-being – responses, intent, plan, etc. while administering the C-SSRS (Columbia Suicidality Severity Response Scale). If these procedures result in any serious concerns on the part of the investigator, they will either ask the participant to meet with or call the PI or escort the participant to the

emergency room. If necessary, they will also call the participant's treating clinician and/or their emergency contact. As part of the consent, participants are required to designate an emergency contact person and provide their treating clinician's contact information. We will ask the treating clinician for their local plan for such emergencies so the study team can follow this plan should an emergency situation arise.

7. Payments for Participation (Economic Considerations)

Participants will not be paid for their participation. Expenses of participating in the study, such as parking will be covered by the study.

8. Assessment of Safety

8.1. Specification of Safety Parameters

8.1.1. Definition of Adverse Events (AE)

Adverse events (AEs) are defined any untoward, physical, social, economic, or psychological occurrence affecting human subjects in research. An AE can be any unfavorable or unintended event including abnormal laboratory finding, symptom, reaction, or disease. An AE does not necessarily have a causal relationship with the research, or any risk associatedwith the research, the research intervention, or the assessment. Adverse events involve subjects only.

8.1.2. Definition of Serious Adverse Events (SAE)

In addition to grading the adverse event, adverse events are evaluated to determine whether they meet the criteria for a Serious Adverse Event (SAE). An SAE is defined as death; a life threatening experience; inpatient hospitalization (for a person not already hospitalized); prolongation of hospitalization (for a patient already hospitalized); persistent or significant disability or incapacity; congenital anomaly and/or birth defects; or an event that jeopardizes the participant and may require medical, surgical, or other intervention to prevent one of the preceding conditions (FDA, 21 CFR Part 312). An adverse event is considered serious if it:

- is life-threatening
- results in in-patient hospitalization or prolongation of existing hospitalization
- results in persistent or significant disability or incapacity
- results in a congenital anomaly or birth defect OR
- results in death
- based upon appropriate medical judgment, may jeopardize the subject's health, and may require medical or surgical intervention to prevent one of the other outcomes listed in this definition, or
- adversely affects the risk/benefit ratio of the study

An adverse event may be graded as severe but still not meet the criteria for a Serious Adverse Event. Similarly, an adverse event may be graded as moderate but still meet the criteria for an SAE. It is important for the PI to consider the grade of the event as well as its "seriousness" when determining whether reporting to the IRBs and the Medical Monitor is necessary.

8.1.3. Definition of Unanticipated Problems (UP)

An unexpected adverse event is one that is not listed in the current Investigators Brochure for Psilocybin, assisted psychotherapy produced by MAPS or an event that is by nature more specific or more severe than a listed event.

Such an event is unlikely because as of 01 December 2016, with over 1000 individuals exposed to Psilocybin in controlled research settings, there have been no unexpected drug-related SAEs to date and expected SAEs have been rare and non-life threatening.

8.1.4. Classification of an Adverse Event

The following scale will be used in grading the severity of adverse events noted during thestudy:

1. Mild adverse event (does not affect patient activity)
2. Moderate adverse event (mild disruption in usual activity)
3. Severe (major disruption in usual activity)

8.2. Time Period and Frequency for Event Assessment and Follow-Up

Adverse events will be reviewed with subjects weekly from the time of preparation visit 1 through primary outcome collect at 4 weeks. Information will be assessed in-person and during weekly phone calls. Adverse events or unanticipated problems involving risks to subjects or others will be reported to the HIC or HSC within 48 hours of it becoming known to the investigator, usingthe appropriate forms found on the website.

8.3. Reporting Procedure (Adverse Events)

The investigator will report the following types of adverse events:

a. serious and unanticipated and possibly, probably, or definitely related events;
b. anticipated adverse events occurring with a greater frequency than expected;
c. other unanticipated problems involving risks to subjects or others.

8.4. Study Halting Rules

The Medical Monitor or the Principal Investigator has the right to discontinue this study at any time. If the trial is prematurely terminated, the Primary Investigator is to promptly inform the study participants and will assure appropriate therapy and follow- up. If the trial or study is prematurely discontinued, all procedures and requirements pertaining to retention and storage of documents will be observed. All other study materials will be treated in accordance with federal and state regulations.

8.5. Attribution of Adverse Events

Adverse events will be monitored for each subject participating in the study and attributed to the study procedures / design by the principalinvestigator according to the following categories:

a. Definite: Adverse event is clearly related to investigational procedures(s)/agent(s).
b. Probable: Adverse event is likely related to investigational procedures(s)/agent(s).
c. Possible: Adverse event may be related to investigational procedures(s)/agent(s).
d. Unlikely: Adverse event is likely not to be related to the investigational procedures(s)/agent(s).
e. Unrelated: Adverse event is clearly not related to investigational procedures(s)/agent(s).

8.7. Plan for Grading Adverse Events: The following scale will be used in grading the severity ofadverse events noted during the study:

a. Mild adverse event
b. Moderate adverse event

c. Severe

8.8. Plan for Determining Seriousness of Adverse Events:
Serious Adverse Events: In addition to grading the adverse event, the PI will determine whether the adverse event meets the criteria for a Serious Adverse Event (SAE). An adverse event is considered serious if it results in any of the following outcomes:

a. Death;
b. A life-threatening experience in-patient hospitalization or prolongation of existing hospitalization;
c. A persistent or significant disability or incapacity;
d. Any other adverse event that, based upon appropriate medical judgment, may jeopardize the subject's health, and may require medical or surgical intervention to prevent one of the other outcomes listed in this definition.

An adverse event may be graded as severe but still not meet the criteria for a Serious Adverse Event. Similarly, an adverse event may be graded as moderate but still meet the criteria for an SAE. It is important for the PI to consider the grade of the event as well as its "seriousness" when determining whether reporting to the IRB is necessary.

8.9. Plan for reporting UPIRSOs (including Adverse Events) to the IRB.
The Principal Investigator will report the following types of events to the IRB: Any incident, experience or outcome that meets ALL 3 of the following criteria:

1. Is unexpected (in terms of nature, specificity, severity, or frequency) given (a) the research procedures described in the protocol-related documents, such as the IRB-approved protocol and informed consent document and (b) the characteristics of the subject population being studied; AND
2. Is related or possibly related to participation in the research (*possibly related* means there is a reasonable possibility that the incident, experience, or outcome may have been caused by the procedures involved in the research); AND
3. Suggests that the research places subjects or others at greater risk of harm (including physical, psychological, economic, legal, or social harm) than was previously known or recognized.

8.10. Unanticipated Problems Involving Risks to Subjects or Others (UPIRSOs) may be medical or non-medical in nature and include – but are not limited to – *serious, unexpected, and related adverse events* and *unanticipated adverse device effects*. *Please note* that adverse events are reportable to the IRB as UPIRSOs only if they meet all 3 criteria listed above.

These UPIRSOs/SAEs will be reported to the IRB using the appropriate forms. Inthe event that such events occur, Reportable Events (which are serious or life- threatening and unanticipated, or anticipated but occurring with a greater frequency than expected, and possibly, probably, or definitely related) or Unanticipated Problems Involving Risks to Subjects or Others that may require a temporary or permanent interruption of study activities will be reported immediately (if possible), followed by a written report to the IRB within 5 calendar days (in accordance with IRB Policy 710) of the Principal Investigator becoming aware of the event (using the appropriate forms) and to any appropriate funding and regulatory agencies.

All related events involving risk but not meeting the *prompt* reporting requirements

should be reported to the IRB in summary form at the timeof continuing review. If appropriate, such summary may be a simple brief statement that events have occurred at the expected frequency and level of severity as previously documented.

8.11. Plan for reporting adverse events to co-investigators on the study, research monitor(s), e.g., Protocol Review Committee (PRC), study sponsors, funding and regulatory agencies, and regulatory and decision-making bodies.

For the current study, the following individuals, funding, and/or regulatory agencies will be notified:

a. All Co-Investigators listed on the protocol.
b. Food and Drug Administration
c. Study Funding and Sponsors

The investigator will apprise fellow investigators and study personnel of all UPIRSOs and adverse events that occur during the conduct of this research project throughregular study meetings, or via email as they are reviewed by the principal investigator.

The protocol's research study sponsors, funding and regulatory agencies, and regulatory and decision-making bodies will be informed of any Serious, Unanticipated and Related adverse events within 5 days (or within any contract or policy designated time period) of the event becoming known to the principal investigator.

The principal investigator will conduct a review of all adverse events upon completion of every study subject. The principal investigator will evaluate the frequency and severity of the adverse events and determine if modifications to theprotocol or consent form are required.

8.12. Safety Oversight

The principal investigator in conjunction with the Medical Monitor will be responsible for monitoring the data, assuring protocol compliance, and conducting the safety reviews at the specified frequency, which must be conducted at aminimum of every 6 months (including when reapproval of the protocol is sought).

During the review process, the principal investigator (in collaboration with the Medical Monitor) will evaluate whether the study should continue unchanged, require modification or amendment, continue or close to enrollment. Either the principal investigator, the HIC, or the Medical Monitor have the authority to stop or suspend the study or require modifications. Likewise, the principal investigator will periodically review the collection, storage, and distribution practices associated with clinical data bank, and determine whether changes to enhance confidentiality and privacy are required. The research team will follow the University of Washington guidelines for attribution of adverse events andgrading the severity of adverse events (including determining whether the adverse event meets criteria for a Serious Adverse Event).

8.13. Clinical Monitoring

After administration of Psilocybin/placebo, participants will be checked regularly. A research team member will accompany the participants and be available at all times tocall for medical assistance if required. Procedures will be immediately stopped if participants request to stop or exhibit any sign of significant distress. The study therapists will have immediate access to the PI or a designated physician to

assess any problems, or to clear a participant for discharge from the research facility.

9. Statistical Considerations

9.1. Measures to Minimize Bias

9.1.1. Enrollment/Randomization/Masking Procedures

The necessary strict exclusion criteria for psilocybin interventions may limit generalizability of the findings. The influence of repeated testing in the crossover design will be minimized by modeling order effects in analyses, but some effects of practice may persist.

9.1.2. Evaluation of Success of Blinding

At the end of the randomized treatment, participants will be asked to fill out a 5 point Likert scale with the following options:

--I am positive I received psilocybin
--I think I received psilocybin
--I cannot tell whether I received psilocybin or niacin (placebo)
--I think I received niacin (placebo)
--I am positive I received niacin (placebo)

9.1.3. Source Documents and Access to Source Data/Documents

Paper source documents and research case books will be housed in restricted access files. Personally-identified information will be restricted to name, address, phone number, birth-date, social security numbers, dates of care, and will be kept by investigators in a separate file from the research data which will be coded using a unique subject identification.

9.1.4. Quality Assurance and Quality Control

The principal investigator is responsible for monitoring the data, assuring protocol compliance, and conducting the safety reviews yearly. During the review process, the principal investigator will evaluate whether the study should continue unchanged, require modification/amendment, continue or close to enrollment. Either the principal investigator or the IRB have the authority to stop or suspend the study or require modifications.

9.2. Statistical approach

The primary objective of this randomized trial is to compare the mean change from baseline MADRS among patients randomized to receive psilocybin to that among patients randomized to receive niacin. The time point of primary interest at which change will be assessed will be 4 weeks. Mean change between the two groups will be assessed with the two-sample t-test, or the Wilcoxon rank-sum test if distributional assumptions for the t-test are violated. The table below shows the minimum detectable assumed-true effect size (represented in standard-deviation units, or the difference in mean change divided by the common standard deviation) to observe a statistically significant difference (at the two-sided significance level of .05) with 80% and 90% power for a variety of total sample sizes.

Sample size	*80% Power*	*90% Power*
**N=30**	1.06 SD units	1.23 SD units
**N=40**	0.91 SD units	1.05 SD units
**N=50**	0.81 SD units	0.94 SD units

Based on the values in the above table, we will randomize 30 patients, as we hypothesize that an assumed-true effect size of 1.06 – 1.23 is achievable based on previous studies by Ross and Davis and achieved effect sizes of 1.46 at 2 weeks at 1.69 at 6 weeks[19] or 2.2 at 5 weeks.[18] **A sample size of N=30 will provide >90% power to detect a significant difference in the primary outcome** between the psilocybin 25 mg vs niacin groups. Assuming a 15% drop-out rate of subjects in this study, we will enroll 36 subjects and assume 6 will not complete the study, so full enrollment in this study will be 36 with a target of 30 subjects completing the study.

The primary analysis will be done using the intent-to-treat principle. Randomization will be done using a permuted block design with randomly selected block sizes.

Key secondary endpoints include those listed below, and a hierarchical approach will be taken to the potential analysis of these endpoints. If the primary endpoint is statistically significant, then these secondary endpoints will be (potentially) formally compared in a hierarchical manner in the order listed below.

1. Mean change in burnout (Stanford Fulfillment index) from baseline to 4 weeks between the two groups
2. Mean change in symptoms of PTSD (PCL5) from baseline to 1 week and 4 weeks between the two groups.

These secondary endpoints will be compared between groups as described above. At the first endpoint that is not statistically significant (at the two-sided significance level of .05), all subsequent endpoints will be evaluated descriptively and not formally assessed in order to maintain the overall type I error rate of .05.

10. Ethics/Protection of Human Subjects

10.1. Ethical Standards

The research will be performed according to ethical principles and in compliance with all federal, state, and local laws, as well as institutional regulations and policies regarding the protection of human subjects.

10.2. Informed Consent Process

After an initial phone screen to determine obvious exclusions from the study protocol, potential subjects will be invited for an in-office visit. During the screening visit, the study procedures will be described, and the subject's questions will be answered prior to obtaining an informed consent. The person obtaining the consent will ask the participant to provide a brief summary of the study to ensure they understand what is being asked of them and anypotential risks and benefits. For participants who are out of the area, informed consent will be obtained via video conference and written consent documented using REDCap.

10.3. Participant and Data Confidentiality

Participant study identification numbers (which cannot be linked to participant), names, and other demographic information, contact information, and previous medical history will be kept in a locked filing cabinet located in a locked room. Secure research files will be used to store assessment and data forms derived from the research data collection. Each participant is assigned a numeric code to conceal identity. This coding will carefully safeguard confidentiality on encrypted computer hard disks used for analysis. No personal identifying data will appear in the computerfiles or other papers (e.g., questionnaires, laboratory raw data).

Care will be taken to conceal other possible sources of identification, including addresses or occupations. A list of participant numbers, names, and other demographic information will be kept in a locked cabinet located in a locked room. Cross-referenced names and code numbers will be included in a master list that will be stored in a locked file cabinet that will be accessible only to the research staff. Theidentities of participants will not be revealed in any description or publications resulting from this research. The granting agency, the research monitors, the PI's laboratory, and collaborators will have access to de-identified study data. All electronic data is double password-protected and follows IRB standards at the University of Washington. Digital data will be stored on a secured server and on secured desktop computers.

10.4. Research Use of Stored Human Data

The investigators understand the importance of communicating research findings from studies. Results from this study may be presented at conferences and meetings or published

in academic journals. All data would be deidentified and participant identity would never be disclosed when presenting results of the study.

10.5. Future Use of Stored Data

De-identified data may be stored indefinitely and used for publications, research presentations and meetings.

11. Data Handling and Record Keeping

11.1. Data Collection and Management Responsibilities

The principal investigator, Anthony Back MD, will be responsible for monitoring the data, assuring protocol compliance, and conducting the safety reviews at the specified frequency, which must be conducted at a minimum of every 6 months. Dr. Back will confer and discuss any issues with the research and oversight team that have been brought to light and will discuss what alterations might be needed if any, in weekly rounds meetings (as is the clinic standard research practice). Any adverse events will be reported in accordance with University of Washington policies.

During the review process, the principal investigator and the Medical Monitor will evaluate whether the study should continue unchanged, require modification/amendment, or close to enrollment. Either the principal investigator, the IRB, or University of Washington administrators have the authority to stop or suspend the study or require modifications.

Research personnel will collect research data via direct interview after potential subjects' sign release of information for available informants. This data will be recorded in both written and/or electronic formats, and all confidential research data will be separated and stored in locked and secured files.

11.2. Study Records Retention

All electronic research records will continue to be encrypted and password-protected after subject participation. Both written and electronic records will be destroyed 10 years after the protocol is completed.

12. Publication and Data Sharing Policy

The identities of participants will not be revealed in any description or publications resulting from this research. The granting agency, the research monitors, the PI's laboratory, and collaborators will have access to de-identified study data.

13. Study Administration and Leadership

13.1. Principal Investigator
Anthony Back MD

14. Conflict of Interest Policy

There is no conflict of interest to report from any of the study staff.

15. References

1. Ouyang H. I'm an E.R. Doctor in New York. None of Us Will Ever Be the Same. The New York Times. New York, NY: The New York Times Company; 2020.
2. Buchbiner L. How doctors' fears of getting COVID-19 can mean losing the healing power of touch: One physician's story. TheConversation.com2020.
3. Choo E. Covid-19 is pushing doctors to the brink. Medicine needs to recognize they're human and need help. The Washington Post. Washington DC2020.
4. Marwa S. A Double Whammy: The COVID-19 Pandemic and Burnout in Medical Professionals. Lean Forward. A place for medical providers to converse on health care topics. Boston, MA: Harvard Medical School; 2020.
5. Jain S. Bracing for an Epidemic of PTSD Among COVID-19 Workers. Psychology Today2020.
6. Mock J, Schwartz J. How Doctors and Nurses Manage Coronavirus Grief. Scientific American2020.
7. Lai J, Ma S, Wang Y, et al. Factors Associated With Mental Health Outcomes Among Health Care Workers Exposed to Coronavirus Disease 2019. JAMA Netw Open 2020;3(3):e203976. DOI: 10.1001/jamanetworkopen.2020.3976.
8. Liu Q, Luo D, Haase JE, et al. The experiences of health-care providers during the COVID-19 crisis in China: a qualitative study. Lancet Glob Health 2020;8(6):e790-e798. DOI: 10.1016/S2214-109X(20)30204-7.
9. Knoll C, Watkins A, Rothfeld M. 'I Couldn't Do Anything': The Virus and an E.R. Doctor's Suicide. The New York Times. New York NY2020.
10. Hendrickson RC, Slevin RA, Chang BP, Sano E, McCall C, Raskind MA. The impact of working during the Covid-19 pandemic on health care workers and first responders: mental health, function, and professional retention. MedRxiv 2021. DOI: doi.org/10.1101/2020.12.16.20248325.
11. Quick COVID-19 Survey. The Larry A. Green Center; 2020.
12. Haugen PT, Evces M, Weiss DS. Treating posttraumatic stress disorder in first responders: a systematic review. Clin Psychol Rev 2012;32(5):370-80. DOI: 10.1016/j.cpr.2012.04.001.
13. Alden L, Matthews LR, Wagner S, et al. Systematic literature review of psychological interventions for first responders. Work & Stress 2020. DOI: DOI: 10.1080/02678373.2020.1758833.
14. Stoesser K, Cobb NM. Self-treatment and informal treatment for depression among resident physicians. Fam Med 2014;46(10):797-801. (https://www.ncbi.nlm.nih.gov/pubmed/25646832).
15. Nutt D, Erritzoe D, Carhart-Harris R. Psychedelic Psychiatry's Brave New World. Cell 2020;181(1):24-28. DOI: 10.1016/j.cell.2020.03.020.
16. Kerbs TS, Johansen P. Over 30 million psychedelic users in the United States. F1000 Research. DOI: https://doi.org/10.12688/f1000research.2-98.v1.
17. Saplakoglu Y. FDA Calls Psychedelic Psilocybin a 'Breakthrough Therapy' for Severe Depression. Live Science2019.

18. Davis AK, Barrett FS, May DG, et al. Effects of Psilocybin-Assisted Therapy on Major Depressive Disorder: A Randomized Clinical Trial. JAMA Psychiatry 2020. DOI: 10.1001/jamapsychiatry.2020.3285.
19. Ross S, Bossis A, Guss J, et al. Rapid and sustained symptom reduction following psilocybin treatment for anxiety and depression in patients with life-threatening cancer: a randomized controlled trial. J Psychopharmacol 2016;30(12):1165-1180. DOI: 10.1177/0269881116675512.
20. Chi T, Gold JA. A review of emerging therapeutic potential of psychedelic drugs in the treatment of psychiatric illnesses. J Neurol Sci 2020;411:116715. DOI: 10.1016/j.jns.2020.116715.
21. A Phase 3 Program of MDMA-Assisted Therapy for the Treatment of Severe Posttraumatic Stress Disorder (PTSD). (https://maps.org/research/mdma/ptsd/phase3).
22. Sessa B, Higbed L, Nutt D. A Review of 3,4-methylenedioxymethamphetamine (MDMA)-Assisted Psychotherapy. Front Psychiatry 2019;10:138. DOI: 10.3389/fpsyt.2019.00138.
23. Griffiths RR, Johnson MW, Carducci MA, et al. Psilocybin produces substantial and sustained decreases in depression and anxiety in patients with life-threatening cancer: A randomized double-blind trial. J Psychopharmacol 2016;30(12):1181-1197. DOI: 10.1177/0269881116675513.
24. Swift TC, Belser AB, Agin-Liebes G, et al. Cancer at the Dinner Table: Experiences of Psilocybin-Assisted Psychotherapy for the Treatment of Cancer-Related Distress. Journal of Humanistic Psychology 2017;57. DOI: 10.1177/0022167817715966.
25. Hasler F, Grimberg U, Benz MA, Huber T, Vollenweider FX. Acute psychological and physiological effects of psilocybin in healthy humans: a double-blind, placebo-controlled dose-effect study. Psychopharmacology (Berl) 2004;172(2):145-56. (In eng). DOI: 10.1007/s00213-003-1640-6.
26. Tyls F, Palenicek T, Horacek J. Psilocybin--summary of knowledge and new perspectives. Eur Neuropsychopharmacol 2014;24(3):342-56. DOI: 10.1016/j.euroneuro.2013.12.006.
27. Moreno FA, Wiegand CB, Taitano EK, Delgado PL. Safety, tolerability, and efficacy of psilocybin in 9 patients with obsessive-compulsive disorder. J Clin Psychiatry 2006;67(11):1735-40. (In eng). DOI: 10.4088/jcp.v67n1110.
28. Passie T, Seifert J, Schneider U, Emrich HM. The pharmacology of psilocybin. Addict Biol 2002;7(4):357-64. (In eng). DOI: 10.1080/1355621021000005937.
29. Nichols DE. Psychedelics. Pharmacol Rev 2016;68(2):264-355. DOI: 10.1124/pr.115.011478.
30. Usdin E, Efron DH. Psychotropic Drugs and Related Compounds. Washington D.C.: National Institute of Mental Health, 1972.
31. COMPASS Pathways and King's College London Announce Results From Psilocybin Study In Healthy Volunteers. 2019.
32. Franz M, Regele H, Kirchmair M, et al. Magic mushrooms: hope for a 'cheap high' resulting in end-stage renal failure. Nephrol Dial Transplant 1996;11(11):2324-27. DOI: 10.1093/oxfordjournals.ndt.a027160.
33. Lim TH, Wasywich CA, Ruygrok PN. A fatal case of 'magic mushroom' ingestion in a heart transplant recipient. Intern Med J 2012;42(11):1268-9. DOI: 10.1111/j.1445-5994.2012.02955.x.
34. Gerault A, Picart D. Intoxication mortelle à la suite de la consommation volontaire et en groupe de champignons hallucinogeèes. Bull Soc Mycol France 1996;112:1-14.
35. Buck RW. Mushroom Toxins — A Brief Review of the Literature. New Engl J Med 1961;265:681-686.
36. Investigational Drug & Material Supply Program. Usona Institute. (https://www.usonainstitute.org/drug-supply/).
37. Muttoni S, Ardissino M, John C. Classical psychedelics for the treatment of depression and anxiety: A systematic review. J Affect Disord 2019;258:11-24. DOI: 10.1016/j.jad.2019.07.076.
38. Bogenschutz MP, Johnson MW. Classic hallucinogens in the treatment of addictions. Prog Neuropsychopharmacol Biol Psychiatry 2016;64:250-8. (In eng). DOI: 10.1016/j.pnpbp.2015.03.002.

39. Daniel J, Haberman M. Clinical potential of psilocybin as a treatment for mental health conditions. Ment Health Clin 2017;7(1):24-28. DOI: 10.9740/mhc.2017.01.024.
40. Johnson MW, Griffiths RR. Potential Therapeutic Effects of Psilocybin. Neurotherapeutics 2017;14(3):734-740. DOI: 10.1007/s13311-017-0542-y.
41. Studerus E, Kometer M, Hasler F, Vollenweider FX. Acute, subacute and long-term subjective effects of psilocybin in healthy humans: a pooled analysis of experimental studies. J Psychopharmacol 2011;25(11):1434-52. DOI: 10.1177/0269881110382466.
42. Carbonaro TM, Bradstreet MP, Barrett FS, et al. Survey study of challenging experiences after ingesting psilocybin mushrooms: Acute and enduring positive and negative consequences. J Psychopharmacol 2016;30(12):1268-1278. DOI: 10.1177/0269881116662634.
43. Johnson M, Richards W, Griffiths R. Human hallucinogen research: guidelines for safety. J Psychopharmacol 2008;22(6):603-20. (In eng). DOI: 10.1177/0269881108093587.
44. Johnson MW, Griffiths RR, Hendricks PS, Henningfield JE. The abuse potential of medical psilocybin according to the 8 factors of the Controlled Substances Act. Neuropharmacology 2018;142:143-166. (In eng). DOI: 10.1016/j.neuropharm.2018.05.012.
45. Treatment Episode Data Set (TEDS) 1994-2004. DEPARTMENT OF HEALTH AND HUMAN SERVICES Substance Abuse and Mental Health Services Administration Office of Applied Studies 2006. (https://wwwdasis.samhsa.gov/dasis2/teds_pubs/2004_teds_rpt.pdf).
46. Griffiths RR, Richards WA, McCann U, Jesse R. Psilocybin can occasion mystical-type experiences having substantial and sustained personal meaning and spiritual significance. Psychopharmacology (Berl) 2006;187(3):268-83; discussion 284-92. DOI: 10.1007/s00213-006-0457-5.
47. Abraham HD, Aldridge AM, Gogia P. The psychopharmacology of hallucinogens. Neuropsychopharmacology 1996;14(4):285-98. DOI: 10.1016/0893-133X(95)00136-2.
48. Mehling WE, Acree M, Stewart A, Silas J, Jones A. The Multidimensional Assessment of Interoceptive Awareness, Version 2 (MAIA-2). PLoS One 2018;13(12):e0208034. DOI: 10.1371/journal.pone.0208034.
49. Hengartner MP, Jakobsen JC, Sorensen A, Ploderl M. Efficacy of new-generation antidepressants assessed with the Montgomery-Asberg Depression Rating Scale, the gold standard clinician rating scale: A meta-analysis of randomised placebo-controlled trials. PLoS One 2020;15(2):e0229381. DOI: 10.1371/journal.pone.0229381.
50. Posner K, Brown GK, Stanley B, et al. The Columbia-Suicide Severity Rating Scale: initial validity and internal consistency findings from three multisite studies with adolescents and adults. Am J Psychiatry 2011;168(12):1266-77. DOI: 10.1176/appi.ajp.2011.10111704.
51. Dozois DJA, Dobson KS, Ahnberg JL. A psychometric evaluation of the Beck Depression Inventory–II. Psychological Assessment 1998;10(2):83-89.
52. Trockel M, Bohman B, Lesure E, et al. A Brief Instrument to Assess Both Burnout and Professional Fulfillment in Physicians: Reliability and Validity, Including Correlation with Self-Reported Medical Errors, in a Sample of Resident and Practicing Physicians. Acad Psychiatry 2018;42(1):11-24. DOI: 10.1007/s40596-017-0849-3.
53. Blevins CA, Weathers FW, Davis MT, Witte TK, Domino JL. The Posttraumatic Stress Disorder Checklist for DSM-5 (PCL-5): Development and Initial Psychometric Evaluation. J Trauma Stress 2015;28(6):489-98. DOI: 10.1002/jts.22059.
54. Barrett FS, Johnson MW, Griffiths RR. Validation of the revised Mystical Experience Questionnaire in experimental sessions with psilocybin. J Psychopharmacol 2015;29(11):1182-90. DOI: 10.1177/0269881115609019.
55. Bond FW, Hayes SC, Baer RA, et al. Preliminary psychometric properties of the Acceptance and Action Questionnaire-II: a revised measure of psychological inflexibility and experiential avoidance. Behav Ther 2011;42(4):676-88. DOI: 10.1016/j.beth.2011.03.007.
56. Garcia-Romeu A, Barrett FS, Carbonaro TM, Johnson MW, Griffiths RR. Optimal dosing for

psilocybin pharmacotherapy: Considering weight-adjusted and fixed dosing approaches. J Psychopharmacol 2021;35(4):353-361. DOI: 10.1177/0269881121991822.
