# Supplementary material for: Psilocybin Therapy for Clinicians With Symptoms of Depression From Frontline Care During the COVID-19 Pandemic: A Randomized Clinical Trial
Source: JAMA Netw Open. 2024 Dec 5;7(12):e2449026. doi: 10.1001/jamanetworkopen.2024.49026 (PMC11621983; doi:10.1001/jamanetworkopen.2024.49026)
Supplement: Supplement 2. — eTable. Functional Unblinding Questions eFigure. Study Flow Diagram [file jamanetwopen-e2449026-s002.pdf]

Supplemental Online Content

Back AL, Freeman-Young TK, Morgan L, et al. Psilocybin therapy for clinicians with symptoms of depression from frontline care during the COVID-19 pandemic: a randomized clinical trial. *JAMA Netw Open*. 2024;7(12):e2449026. doi:10.1001/jamanetworkopen.2024.49026

eTable. Functional Unblinding Questions

eFigure. Study Flow Diagram

This supplemental material has been provided by the authors to give readers additional information about their work.

© 2024 Back AL et al. *JAMA Network Open*

eTable. Functional Unblinding Questions

"Which treatment do you believe you received: the psilocybin or the niacin?"
(just after capsule was swallowed)

**Group**	"psilocybin"	"niacin"	"don't know"
**Psilocybin**	0	0	15 (100%)
**Placebo**	0	0	15 (100%)

"Which treatment do you believe you received: the active drug or the placebo?"
(asked just before discharge from the psilocybin or placebo session)

**Group**	"psilocybin"	"niacin"	"don't know"	Correct at unblinding
**Psilocybin**	0	0	15 (100%)	15 (100%)
**Placebo**	0	0	15 (100%)	15 (100%)

© 2024 Back AL et al. *JAMA Network Open*

eFigure. Study Flow Diagram

Figure legend. The days are numbered using Day 0 as the day of the psilocybin or niacin session. Prep = Preparation session. Integ = Integration session. Baseline measures were completed just before Prep 1.

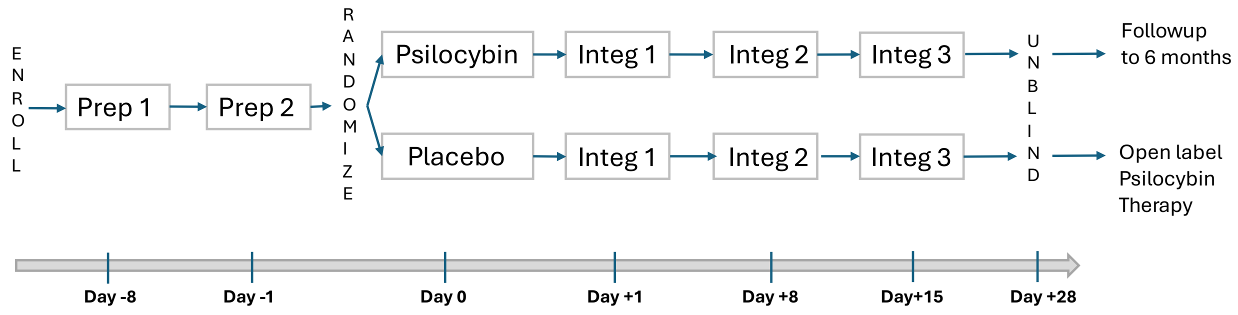

© 2024 Back AL et al. *JAMA Network Open*
