# Supplementary material for: Psilocybin Therapy for Clinicians With Symptoms of Depression From Frontline Care During the COVID-19 Pandemic: A Randomized Clinical Trial
Source: JAMA Netw Open. 2024 Dec 5;7(12):e2449026. doi: 10.1001/jamanetworkopen.2024.49026 (PMC11621983; doi:10.1001/jamanetworkopen.2024.49026)
Supplement: Supplement 3. — Data Sharing Statement [file jamanetwopen-e2449026-s003.pdf]

Data Sharing Statement

Back. Psilocybin Therapy for Clinicians With Symptoms of Depression From Frontline Care During the COVID-19 Pandemic. *JAMA Netw Open*. Published December 05, 2024. doi:10.1001/jamanetworkopen.2024.49026

Data
Additional Information: NCT05163496
Data available: Yes
Data types: Deidentified participant data
How to access data: tonyback@uw.edu
When available: beginning date: 01-01-2025

Supporting Documents
Document types: None

Additional Information
Who can access the data: Researchers whose proposed use of the data has been approved
Types of analyses: For a specified purpose
Mechanisms of data availability: After approval of a proposal
Any additional restrictions: None
